# Advances in Polyurethane-Modified Asphalt via the Prepolymer Method: Molecular Design, Modification Mechanisms, Structural Evolution, and Performance Optimisation

**DOI:** 10.3390/polym18151803

**Published:** 2026-07-23

**Authors:** Haoran Sheng, Rui Ma, Yiming Li, Peifeng Cheng, Aoting Cheng

**Affiliations:** 1 Aulin College, Northeast Forestry University, Harbin 150040, China; henryhrsheng@gmail.com; 2 School of Civil Engineering and Transportation, Northeast Forestry University, Harbin 150040, China; mrui@nefu.edu.cn (R.M.); chengpeifeng2024@163.com (P.C.); chengaoting@nefu.edu.cn (A.C.); 3 School of Transportation, Southeast University, Nanjing 210096, China

**Keywords:** polyurethane prepolymer, modified asphalt, reactive modification, microstructural evolution, rheological properties, storage stability, pavement performance

## Abstract

During long-term service, asphalt pavements undergo environmental stress and ageing, which cause cracking, rutting, and other distresses and raise maintenance costs. Polyurethane (PU) has high mechanical strength, elastic recovery, and ageing resistance due to its unique molecular structure. As an asphalt modifier, PU has been reported to improve high-temperature stability, moisture resistance, and durability. However, PU and asphalt differ greatly in polarity, density, viscosity, and phase structure, and these differences often lead to segregation and phase separation. The prepolymer method can mitigate these compatibility limitations by adjusting molecular weight, terminal-group activity, and soft/hard segment ratio before dispersion, chain extension, crosslinking, and post-curing in asphalt, resulting in better compatibility and more controllable processing. This review discusses PU soft/hard segment structures, asphalt composition, prepolymer synthesis and curing, microstructural evolution, pavement performance, storage stability, and use in other systems to clarify modification mechanisms and potential applications. This critical review aims to clarify material–reaction–process–performance relationships within the prepolymer route, with scope limited to molecular design, preparation mechanisms, performance, storage stability, and representative engineering applications. Future work should consider real service conditions and build multiscale evaluation frameworks that jointly optimise prepolymer design, processing, storage stability, and pavement performance, helping translate laboratory findings into low-carbon, long-life road materials that can be produced at scale.

## 1. Introduction

As motor-vehicle traffic continues to increase, asphalt binder remains widely used in pavements because it is relatively low-cost [1]. Its long-term performance, however, is constrained by short service life, accumulated distress, and maintenance cost. The literature reports that the design life of asphalt pavements on expressways and class I highways in China is 15 years, compared with 30–50 years commonly adopted in developed countries [2]. During service, volatilisation, oxidation, and physical hardening make the asphalt binder brittle, which promotes cracking and shortens fatigue life [3]. Strong ultraviolet ageing can also reduce moisture stability, fracture energy, and fatigue performance [4]. These losses have a direct cost: the equivalent uniform annual cost (EUAC) of structural overlays can reach US$1774–3452 per lane-kilometre [5]. Lower life-cycle maintenance costs therefore depend on optimised maintenance strategies based on life-cycle cost analysis (LCCA) [6], together with material-level approaches that slow ageing and distress development.

Polymer-modified asphalt binders have attracted attention for this reason. The rationale is that polymer chains can adjust asphalt binder viscoelasticity, component mobility, and interfacial structure, improving resistance to rutting, cracking, and fatigue. PE, PS, SBR, and SBS can change diffusion, viscosity, and high-temperature stability in asphalt binder systems [7]. Functionalised PE improves storage stability and deformation resistance [8,9], and low-dosage SBS can enhance basic asphalt binder properties and extend mixture fatigue life [10]. These findings show that polymer modification can improve long-term service performance, although its effectiveness depends on polymer structure, asphalt binder composition, and compatibility between the two.

Bitumen additives can be classified by both chemistry and function. Physical modifiers include thermoplastics (e.g., polyethylene and polypropylene), elastomers, and thermoplastic elastomers such as SBS, whereas chemical modifiers include sulfur, polyphosphoric acid, maleic anhydride, thermosetting resins, and reactive polymers [11,12,13]. Plasticisers and rejuvenators restore flow and low-temperature relaxation by replenishing or mobilising the maltene phase, while hydrocarbon extenders, bio-oils, and waste-derived oils can partially substitute petroleum-derived binder fractions [11,14]. Adhesion promoters and anti-stripping additives form a separate functional group because they are selected primarily to improve binder–aggregate affinity and moisture resistance [11,15]. This taxonomy distinguishes additives that modify bulk rheology from those that replace binder fractions or target interfaces, providing the broader context for the reactive PU route considered here.

Polyurethane (PU) and its derivatives are considered promising asphalt binder modifiers because their soft/hard segment structures are tunable and they offer high mechanical strength, wear resistance, ageing resistance, and potential compatibility with recycled materials [16,17,18,19]. PU, thermoplastic polyurethane (TPU), thermosetting polyurethane (TS-PU), polyurethane prepolymer (PUP), and PU-based composite systems can improve, to varying degrees, high-temperature rutting resistance, low-temperature cracking resistance, fatigue performance, ageing resistance, and storage stability [17,20,21,22,23,24]. In bridge-deck paving, for example, TS-PU-modified asphalt binder has better high-temperature rutting resistance and mechanical strength than SBS-modified asphalt binder and can be less costly than epoxy asphalt [20]. Composite systems such as PU/rock asphalt and RA/TPU improve high- and low-temperature performance and long-term durability through interactions between soft and hard components [21,23]. Photoresponsive self-healing PUPs add polymer reinforcement and crack self-healing, increasing crack-healing efficiency, cracking resistance, and rutting resistance in asphalt mixtures, which may reduce the frequency of later repair [24].

Compatibility still limits the use of PU and its derivatives in asphalt binder. Asphalt binder is a complex colloidal system composed of saturates, aromatics, resins, and asphaltenes. PU/TPU is usually more polar and contains soft/hard segment microphase structures, so differences in composition, polarity, density, and viscosity can produce non-uniform dispersion, aggregation, phase separation, or reduced storage stability [16,25,26,27]. PU–asphalt binder compatibility depends on shear temperature, PU dosage, and molecular structure. At suitable temperatures and dosages, PU can form relatively stable networks through hydrogen bonding, van der Waals interactions, and reactions between isocyanate groups and reactive groups in the asphalt binder. When the PU/TPU dosage is excessive, however, the surplus polymer is difficult to embed fully in the asphalt binder system. It often appears as aggregation or flocculation in fluorescence micrographs; the top-bottom softening-point difference increases, and high-temperature storage stability and compatibility decline [25,26]. Controlling compatibility, dispersion, and storage stability while improving pavement performance remains a main challenge for PU-modified asphalt binder [25,26,27].

Researchers have improved compatibility through compatibilisers, inorganic nanomaterials, and processing control. Organomontmorillonite, for example, restricts the migration of TPU, asphaltenes, and light components through its layered silicate structure. This improves the storage stability of TPU-modified asphalt binder and also strengthens high- and low-temperature performance [28]. Nano-TiO_2_ can form a relatively stable modification network through physical bridging, and ultraviolet shielding and can slow ageing deterioration [29]. In PU/organic attapulgite composite modification, reactive compatibilisers such as maleic anhydride increase asphalt binder polarity and reduce the solubility-parameter difference between PU and asphalt binder, whereas organic attapulgite lowers the risk of phase separation during hot storage through adsorption, barrier effects, and improved composite-phase dispersion [30]. PU molecular structure, free NCO content, post-treatment, and processing also affect interactions between PU and polar asphalt binder components. Compatibility is therefore governed by additive selection, reactivity, and structure formation [31,32].

These compatibility constraints have led many studies to use a prepolymer route for polymer-modified asphalt binders. Rather than mixing all components in one step, this route separates polymer synthesis into prepolymerisation and subsequent reaction stages [33,34,35,36]. As asphalt binder modifiers, PUPs have two practical advantages. They are usually liquid, which allows uniform mixing with asphalt binder at relatively low processing temperatures, such as 90 C, and reduces energy use and processing difficulty. In addition, small amounts of prepolymer can markedly improve asphalt binder rheology, helping to control production cost and improve the competitiveness of modified asphalt binder products [36].

**Figure 1:**
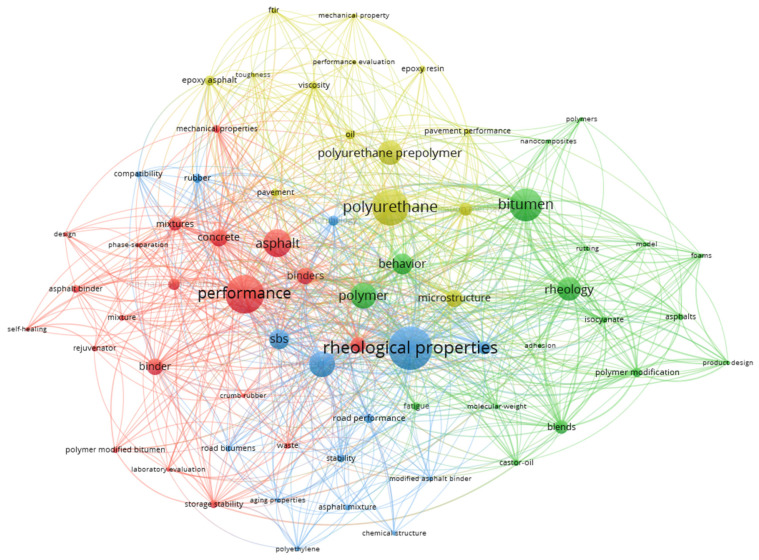
Network diagram of research hotspots in polyurethane prepolymer and asphalt.

Figure 1 shows the keyword co-occurrence network for studies on polyurethane prepolymers and asphalt/bitumen. The largest nodes are asphalt, bitumen, performance, rheological properties, polyurethane, and polyurethane prepolymer, suggesting that current research is centred on the rheological response of asphalt binder and pavement performance. Keywords such as microstructure, adhesion, polymer modification, fatigue, ageing properties, and storage stability show a gradual shift from basic performance evaluation to mechanism-oriented work on phase morphology, interfacial interactions, durability, and construction stability. The network supports the organisation of this review around molecular design, synthesis, and curing mechanisms; microstructural evolution; performance regulation; storage stability; and applications in recycled or composite asphalt binder systems.

Existing reviews mainly catalogue PU types and laboratory performance, but seldom integrate prepolymer molecular design, competing interaction mechanisms, processing and storage constraints, and field implementation. This critical review therefore links these dimensions to identify mechanistic disagreements and barriers to engineering translation.

**Figure 2:**
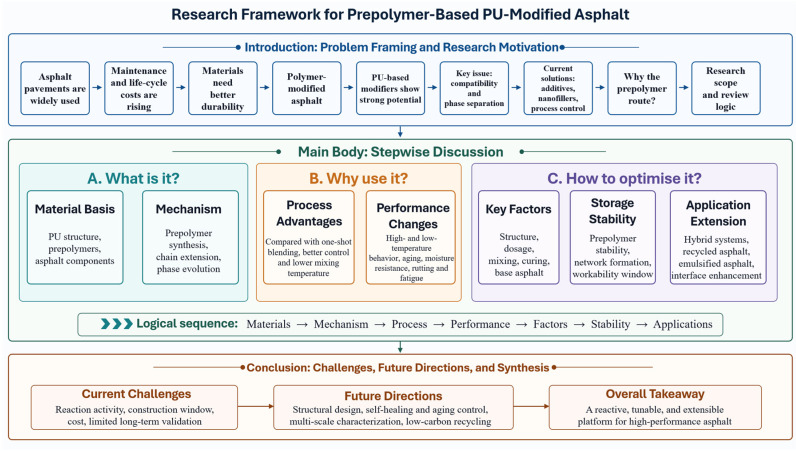
Framework of the paper.

This review summarises progress in the prepolymer method for asphalt binders modified with PU and its derivatives. It focuses on synthesis mechanisms and microstructural evolution, performance changes in modified asphalt binders, prepolymer storage stability, and applications that combine the prepolymer method with recycled or non-traditional asphalt binder systems. The review also identifies unresolved issues and future directions, with the aim of supporting translation of prepolymer-method PU-modified asphalt binder from laboratory research to road-engineering applications. Figure 2 presents the conceptual framework of this review, linking synthesis, structure, performance, storage stability, and application scenarios.

## 2. Material Basis and Reactive Components of Prepolymer Method Polyurethane-Modified Asphalt Binders

### 2.1. Molecular Structure and Reactive-Component Basis of Polyurethane Prepolymers

Polyurethane (PU) modifies asphalt binder mainly through urethane bonds and soft/hard segment structures. Soft segments are usually derived from polyether, polyester, or polycarbonate polyols and control low-temperature flexibility, segmental mobility, and compatibility with light components in asphalt binder. Hard segments, formed from isocyanates and chain extenders, can act as physical crosslinking points through urethane/urea hydrogen bonding and affect strength, modulus, microphase separation, and network stability [37,38].

**Figure 3:**
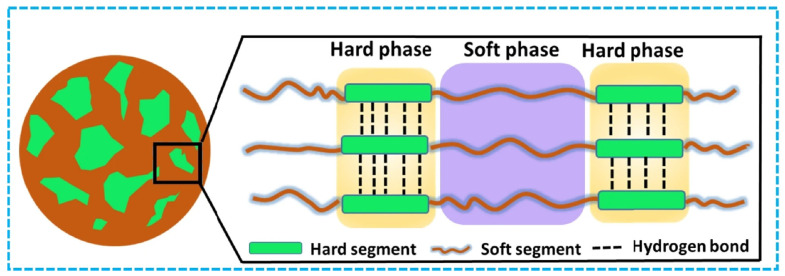
Illustration of PU soft segment and hard segment [39].

Figure 3 shows the molecular-level composition of soft and hard segments in PU.

Isocyanates, polyols, and chain extenders jointly determine PU structure. Isocyanate structure, polyol type and molecular weight, chain-extension/crosslinking components, NCO/OH ratio, free NCO content, and soft/hard segment ratio shape prepolymer viscosity, terminal-group reactivity, hydrogen-bond organisation, phase separation, and dispersion and curing behaviour in asphalt binder [40,41,42,43]. In PU-modified asphalt binder, these raw-material variables also influence PU–asphalt binder compatibility, swelling behaviour, dispersion morphology, and rheological response [40,43]. Table 1 lists the structural formulae and chemical names of common raw materials used in PU synthesis.

Unlike direct addition of thermoplastic PU or in situ formation of thermosetting PU in asphalt binder, a PUP is a reactive intermediate that already contains urethane segments and usually retains terminal -NCO groups. It first forms a controllable oligomeric structure outside the asphalt binder, then continues to grow after entering the binder through chain extension, crosslinking, moisture-induced urea formation, or local reactions with active hydrogen sites. This sequence connects PU molecular design with the evolution of asphalt binder phase structure [44]. Gong et al. compared the prepolymer method with in situ polymerisation for CO_2_-based PU-modified asphalt binder. In the prepolymer method, a PPC-PU prepolymer was prepared first and then added to the asphalt binder for ambient curing, whereas in situ polymerisation involved adding BDO to the asphalt binder for chain extension, which produced more hard segments and a denser crosslinked network [44]. Both modified asphalt binders cured gradually under ambient conditions, and their curing degrees exceeded 80% after 15 days. The in situ polymerisation method gave stronger high-temperature performance because it produced more hard segments and crosslinks, whereas the prepolymer method had a lower Tg and better low-temperature performance because of its higher soft-segment fraction [44].

**Figure 4:**
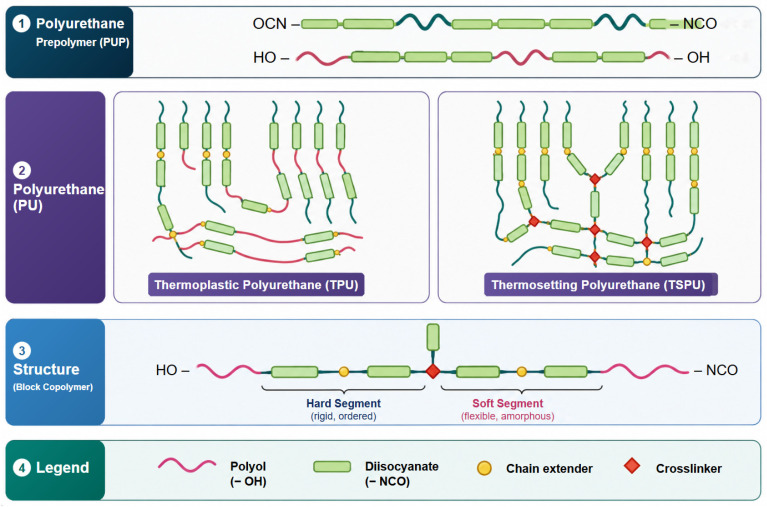
Schematic diagram of the molecular structures of prepolymer, thermosetting, and thermoplastic polyurethane.

Figure 4 schematically shows the structural differences among PUPs, thermosetting PU, and thermoplastic PU [45].

### 2.2. Chemical Composition and Structural Features of Asphalt Binder

Asphalt binder is a complex medium for PUP modification. The SARA fractions provide a useful framework for describing its chemical composition: saturates and aromatics mainly form the low-polarity light phase, resins help stabilise or coat asphaltenes, and asphaltenes contain structures with higher polarity and stronger association capacity. SARA, however, is a solubility- and chromatography-based classification rather than a set of well-defined molecular compositions [46]. Using NMR, FTIR, fluorescence spectroscopy, and elemental analysis, Werkovits et al. further showed that aromatic condensation, heteroatom content, and hydroxyl/amino-containing structures change as component polarity increases from saturates to asphaltenes [47].

Polar functional groups in asphalt binder are especially relevant to PUP modification. Hydroxyl, phenolic hydroxyl, carboxyl, carbonyl, sulfoxide, and nitrogen-containing structures are mainly enriched in highly polar fractions such as resins and asphaltenes. Through hydrogen bonding, acid–base interactions, and polar adsorption, they can influence PU phase dispersion, interfacial adhesion, and ageing sensitivity. Small amounts of active-hydrogen-containing groups may also react locally with terminal NCO [47]. Although these functional groups are generally present at low contents, they can alter dipole moments, hydrogen-bonding capacity, and acid–base interactions in asphalt binder molecules, affecting asphaltene aggregation, interfacial adhesion, and the dispersion environment of added modifiers.

The aggregation state of asphaltenes sets the compatibility environment that prepolymers encounter after entering the asphalt binder. Aromatics and resins help maintain asphaltene dispersion. Loss of light components, oxidation, or addition of reactive components can shift the polarity balance and drive asphaltenes from a dispersed state towards aggregation, which increases stiffness and weakens low-temperature toughness. In the prepolymer method, dispersion, curing, and network formation all occur within this colloidal structure [48]. By analysing linear viscoelastic behaviour using SARA ratios and FTIR polarity indices, Xiao et al. found that saturates and aromatics mainly contribute to viscosity, asphaltenes markedly enhance elasticity, and SARA-fraction polarity strongly correlates with viscoelastic model parameters [49]. Asphalt binder compatibility, therefore, depends on asphaltene content, resin dispersing capacity, aromatic solvency, and the balance among polar fractions.

The source of the base asphalt binder, SARA ratio, initial functional-group content, and ageing degree jointly determine how PUP modifies the asphalt binder. The light phase affects swelling and dispersion, highly polar fractions provide interfacial interactions and potential reaction sites, and asphaltene aggregation influences network continuity and storage stability. In PU systems, these functional groups may also participate in hydrogen bonding, dipolar interactions, or isocyanate-related reactions, affecting PU–asphalt binder compatibility [27]. Ageing of the base asphalt binder changes light-component content, asphaltene aggregation, and the distribution of oxygen-containing functional groups [50,51,52,53,54,55,56,57], which further alters the dispersion, compatibility, and curing environment of PU modifiers. Evaluation of PU-modified asphalt binders should therefore consider SARA ratios, polar functional groups, asphaltene aggregation state, and ageing degree when discussing the material basis.

Engineering-scale evidence also indicates that the constituent package must be selected as a processable system rather than as isolated reactants. In the G18 Rongwu Expressway maintenance project, Li et al. used a low-viscosity, NCO-functional PU prepolymer together with 1.5 wt% SBS in an AC-20 intermediate-course mixture [58]. This application demonstrates the practical relevance of coordinating prepolymer reactivity, binder composition, viscosity, and aggregate compatibility for field production.

## 3. Synthesis Mechanisms and Morphological Evolution of Prepolymer Method PU-Modified Asphalt Binders

The prepolymer method relies on controlling PU structural composition under defined reaction conditions. Isocyanates, polyols, and chain-extension/crosslinking components form urethane bonds, urea bonds, hydrogen bonds, and continuous polymer phases under the influence of temperature, NCO/OH ratio, free NCO, soft/hard segment ratio, and shear history. Preforming oligomers that contain urethane bonds and terminal NCO reduces the uncertainty of direct monomer reactions. Subsequent reactions of residual NCO with chain-extension/crosslinking components then allow continued molecular-weight growth, gelation, and phase-structure rearrangement in asphalt binder [31,44,59,60,61].

### 3.1. Chemical Synthesis Mechanisms of Prepolymers

PUPs are commonly formed by reacting excess diisocyanate or polyisocyanate with oligomeric polyols, giving backbones that contain urethane bonds and retain -NCO groups at chain ends or pendant termini. This approach separates soft-segment formation from later chain extension/crosslinking into two relatively controllable stages, which allows adjustment of initial molecular weight, viscosity, and functionality. Carrera et al. showed that the molecular weight and free NCO content of MDI-PPG terminal-NCO prepolymers jointly determine their capacity for structural growth [31]. Gong et al. distinguished the prepolymer modification route from the BDO in situ chain-extension route for PPC-PU [44]. The PUP used by Sun et al. was synthesised from polyether polyol and 4,4′-MDI, forming a low-molecular-weight intermediate with terminal NCO that, together with chain extenders and asphalt binder, constituted the PPB system [60].

At the reaction level, prepolymer formation mainly proceeds through nucleophilic addition between -NCO and -OH groups to generate -NHCOO- urethane bonds. When diisocyanate is used in excess, some -NCO groups remain as terminal groups. After BDO, TMP, amine chain extenders, or other active-hydrogen components are added, terminal NCO can continue to form urethane or urea structures. Moisture in asphalt binder and polar active hydrogens, including hydroxyl, carboxyl, phenolic hydroxyl, and amine groups, may also induce urea formation, limited grafting, or interfacial reactions [31,44,59,61]. Yang et al. observed by FTIR that the NCO peak decreased during curing, whereas urethane carbonyl and C-O absorption peaks increased, showing that PU curing in asphalt binder remains dominated by the NCO-OH reaction [59]. Kinetic studies show that conversion is relatively fast during the first 120 min but slows after conversion reaches approximately 0.8 because group concentration falls and the developing network restricts mobility [61].

Prepolymer structure is determined by the NCO/OH ratio, free NCO content, polyol molecular weight, isocyanate structure, and chain extenders. Carrera et al. found that MDI-PPG940 prepolymers showed a stronger modification effect at 17.4 wt% free NCO, but the highest NCO content was not necessarily optimal. Molecular size and the number of reactive NCO groups, therefore, need to be balanced [31]. Jin et al. demonstrated that hard-segment content and isocyanate index alter PU aggregation and dispersion morphology in asphalt binder [62], whereas Gallu et al. showed that chain extenders such as BDO, isosorbide, and EHDO affect PU phase separation, crystallinity, and swelling behaviour [40]. In practice, the prepolymer method sets the later curing pathway through terminal groups, soft-segment length, and the mode of hard-segment formation.

Prepolymer design also requires molecular-level characterisation because structure cannot be inferred from average NCO content alone. Blaj et al. used MALDI MS to distinguish unreacted PEG, monofunctionalised PEG, and difunctionalised PEG in PEG-IPDI prepolymers, showing that temperature, concentration, and DBTDL dosage alter the proportion of NCO-functionalised oligomers [63]. The same NCO/OH ratio may therefore correspond to different functionality distributions and phase morphologies. Fage et al. further noted that MIR/NIR can quantitatively track isocyanate content and that PU synthesis is sensitive to stoichiometric ratio, temperature, and residual moisture [64]. Attenuation of the NCO peak in FTIR, formation of urethane carbonyl groups, and, where necessary, NCO titration or spectroscopic quantification should therefore be used as control criteria for prepolymer synthesis.

**Figure 5:**
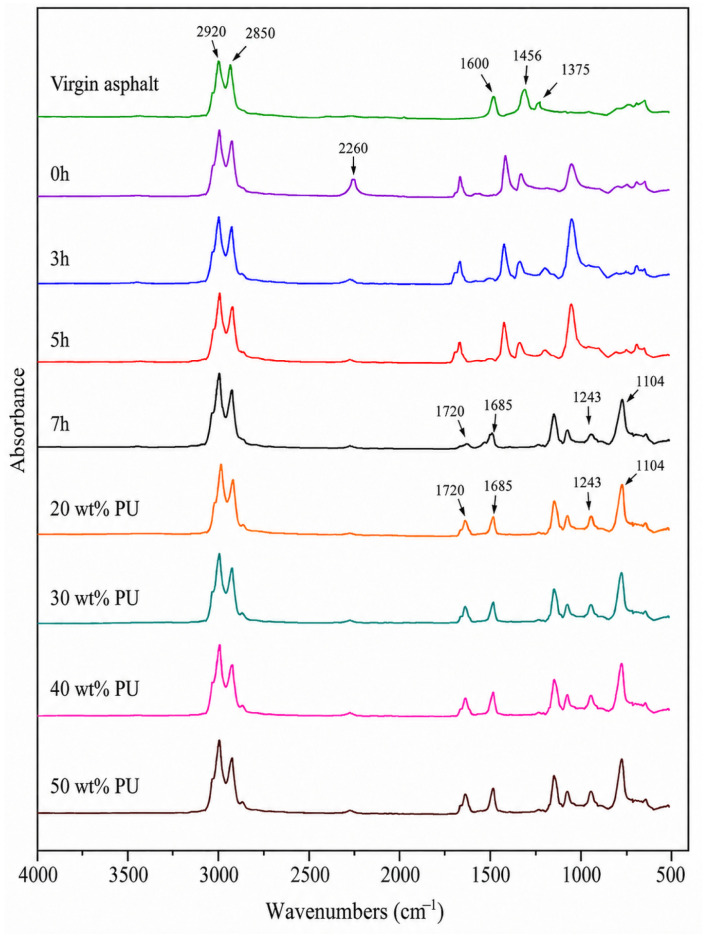
Infrared spectra of virgin asphalt and TPUA samples with different curing times and PU contents.

Figure 5 shows that, during the curing of thermosetting PU in asphalt binder, the characteristic NCO peak decreases and C=O and C-O absorption peaks gradually appear. After 7 h of curing, the 2260 cm^−1^ absorption peak has essentially disappeared in samples with different PU contents [59]. Curing time should therefore be determined from NCO consumption together with viscosity growth, rather than set empirically alone.

Raw material source also affects prepolymer reactions. Bio-based or CO_2_-based polyols still follow the basic pathway in which NCO-OH reactions form urethane bonds, but changes in soft-segment polarity, hydroxyl value, viscosity, and molecular weight alter reaction rate and phase structure [65,66]. Xia et al. synthesised terminal-NCO V-PU from castor oil and liquefied MDI-100LL and observed phase inversion near 30 wt% V-PU [66]. Gong et al. found that CO_2_-based PPC-PU prepolymers can continue to cure under ambient conditions and exceed 80% curing after 15 d, but do not cure completely because some NCO is encapsulated by asphalt binder components and becomes difficult to react further [44].

In practical preparation, the prepolymer method is also affected by shear temperature, shear rate, shear time, and feed scale. Using response surface methodology, Yang et al. proposed an improved combination of PUP amount, shear rate, temperature, and time for PUP-modified asphalt binder [67]. Increasing temperature or extending shear time does not necessarily improve the system. Insufficient shear leads to poor dispersion, whereas excessive temperature or time may accelerate curing, increase viscosity, and shorten the construction window. Sun et al. also found that higher PUP and chain-extender contents accelerate viscosity growth in PPB, with some formulations reaching 5000 mPa s too rapidly for construction retention time [60]. Prepolymer synthesis should therefore account for both chemical composition and processing parameters. The key structural variables and their material significance are summarised in Table 2.

### 3.2. Microstructural Changes During Synthesis by the Prepolymer Method

When PU-modified asphalt binder is prepared by the prepolymer method, microstructural evolution first depends on how the reactive oligomer enters the system. Compared with preformed thermoplastic PU particles, terminal-NCO prepolymers, MDI-PEG/MDI-PPG reactive prepolymers, or PUP have lower molecular weight and better fluidity during early mixing. They can disperse and swell in the continuous asphalt binder phase and contact polar microdomains before chain extenders, moisture, and active sites in the asphalt binder promote formation of urethane, urea, amide, or higher-molecular-weight structures [31,32,68,69,70,71,72].

Studies of early MDI-PEG and MDI-PPG systems show how this structural evolution occurs. Navarro et al. reported that low-viscosity isocyanate-terminated prepolymers may initially plasticise asphalt binder; during curing at room temperature or 90 degrees C, however, residual NCO reacts with polar compounds in the asphalt binder, gradually increasing the linear viscoelastic modulus and viscosity [68]. Martin-Alfonso et al. used rheology, thermal analysis, and AFM to show that MDI-PEG can increase viscosity at 60 degrees C over curing periods longer than one week and form new microstructures associated with polar components [69]. Moisture and temperature affect post-curing and short-term reaction kinetics in different ways: moisture promotes further residual NCO reaction, low temperature favours slow torque growth, and high temperature changes both the reaction rate and final structure [70,71].

Prepolymer structural parameters govern the subsequent formation of phase structures. Carrera et al. compared PPG molecular weights, free NCO contents, and water-addition conditions, and found that a prepolymer with a molecular weight of approximately 940 and 17.4 wt.% free NCO produced the strongest modification after water addition, although the asphalt binder source strongly affected efficiency [31]. Their colloidal-property study further showed that asphalt binders with higher polar-component contents and more developed asphaltene colloidal structures were more likely to form isocyanate-prepolymer-induced three-dimensional networks [72]. The resulting microstructure is therefore controlled jointly by NCO reactivity, polar components in the asphalt binder, and colloidal microstructure.

Processing conditions determine whether the prepolymer forms a uniform dispersion before curing. Yang et al. identified interactions among preparation amount, shear rate, temperature, and time; 6–10% PUP balanced conventional indices, toughness, storage stability, and viscosity, and 8% PUP gave better high-temperature deformation resistance [67]. The low-viscosity advantage of the prepolymer is retained only under suitable shear and temperature conditions. Rapid reaction, excessive viscosity, or poor compatibility can reduce that advantage.

Li et al. combined graphical and textual characterisation to examine the in situ formation of PU from PU prepolymer and chain extender in asphalt binder. FTIR, component separation, confocal fluorescence microscopy, particle-size distribution, rheology, and theoretical calculations showed that PU has little effect on the bulk chemistry of asphalt binder and acts mainly through physical dispersion; the in situ generated PU was relatively uniformly distributed, its particle size followed a log-normal distribution, and particles became markedly larger when PU content exceeded 15% [73].

**Figure 6:**
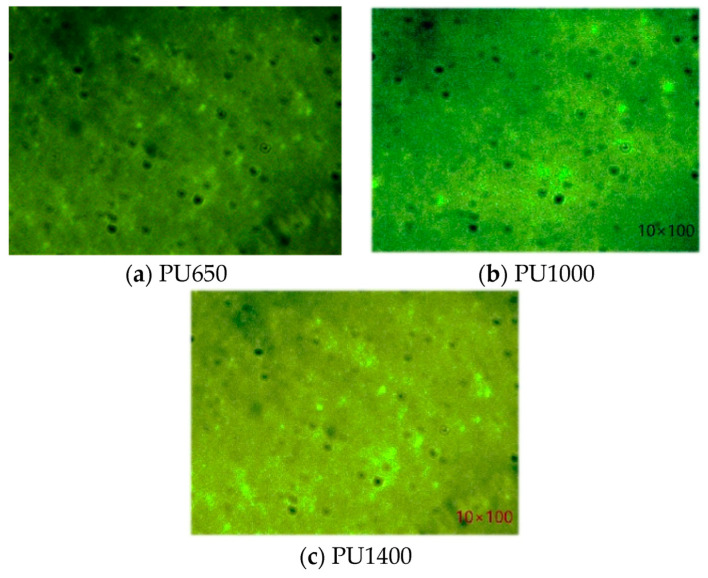
Fluorescence microscopy test: (**a**) PU650-modified asphalt, (**b**) PU1000-modified asphalt, and (**c**) PU1400-modified asphalt [74].

Figure 6 shows fluorescence microscopy images of asphalt binders modified with PU prepolymers of different molecular weights [74].

**Figure 7:**
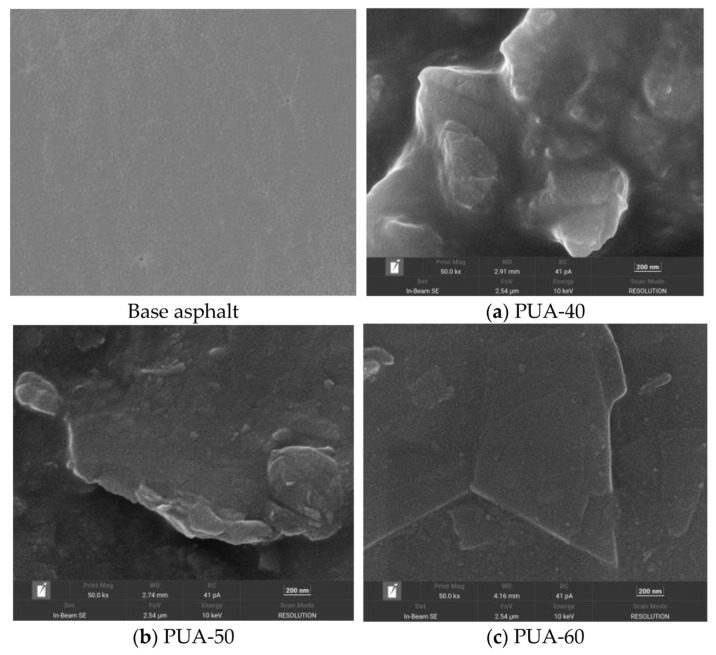
SEM images of thermosetting PU-modified asphalt with different PU dosages [75].

The SEM images in Figure 7 further show the morphology changes associated with PU content. The base asphalt binder shows no continuous network. PUA-40 has protrusions and a relatively clear PU–asphalt binder interface, indicating formation of a PU phase that is not yet well integrated. In PUA-50, protrusions and interfacial separation are reduced. In PUA-60, the surface is smoother, and the interface tends to merge, consistent with the gradual formation of a continuous PU phase and crosslinked network [75].

In prepolymer synthesis, chain extenders substantially change the compactness of the PU network. Gong et al. prepared P-PUMA and I-PUMA using CO_2_-based PPC-PU prepolymers; FTIR and TGA confirmed good thermal stability, and the modified asphalt binder cured naturally, with a curing degree above 80% after 15 d [44]. P-PUMA mainly depends on the gradual reaction of terminal NCO with environmental moisture and active groups in the asphalt binder, so network formation is slower. I-PUMA introduces more hard segments and junction points through BDO chain extension, forms a denser network, and shows property improvements with curing time [44].

**Figure 8:**
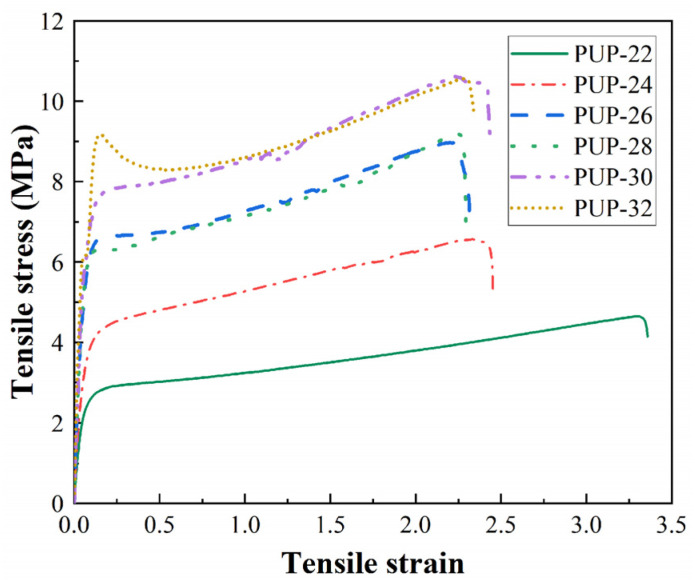
The tensile stress–strain curves of PUP samples [60].

Figure 8 shows that increasing the chain-extender proportion shifts the stress–strain curve upward and increases material strength and stiffness, while ductility gradually decreases. The PU network, therefore, changes from a relatively flexible structure to a denser and more highly crosslinked one. Chain extenders can strengthen the PU network and improve the load-bearing capacity of the modified system [60].

PUPB and PPB systems show that the prepolymer method can drive structural evolution from particle dispersion to crosslinked gelation. Sun et al. synthesised PUP with an NCO content of 10.20–11.20% using polyether polyol and 4,4′-MDI, and prepared PPB with MDBA and maleic anhydride. Viscosity–time curves showed slow thickening at the early stage followed by marked acceleration after approximately 30 min, with PUP content controlling the reaction rate more strongly than the chain extender [60]. Tensile results showed that higher PUP content increased strength but reduced elongation at break, indicating that the PUP-MDBA skeleton improves strength at the expense of flexibility when PUP is excessive [60].

In a PUPB/FRAP system, Wang et al. related this evolution to molecular weight, chemical bonds, and interfaces. FTIR and GPC showed that nitrogen-containing and carbon-containing structures, macromolecular fractions, and molecular weight in PUPB increased with PUP content. Crosslinking polymerisation of PUP resin was the dominant process, together with interactions between a small amount of PUP and active sites in asphaltenes [76]. The network improved high-temperature and thermo-oxidative ageing stability, but the soft segments provided limited improvement in low-temperature cracking resistance, so network reinforcement and flexibility still need to be balanced [76].

Functionalised prepolymers can also form self-healing, reactive regeneration, or fibre-composite networks. Shirzad et al. developed a UV-triggered self-healing PU prepolymer; FTIR confirmed urethane-bond formation and residual NCO consumption. The system improved high-temperature grade, elastic recovery, and crack-healing ability, but healing efficiency decreased when SPP increased from 10% to 15%, showing that excessive prepolymer content may weaken the cohesive structure [24,77]. In the PUP/ceramic fibre system reported by Guo et al., the PUP gel network filled CF–asphalt binder gaps and bridged weak interfaces, forming a composite support structure comprising PUP, fibres, and asphalt binder [78].

Taken together, the microstructural evolution of PU-modified asphalt binder prepared by the prepolymer method can be described as four continuous processes. First, the low-viscosity prepolymer disperses and contacts polar microdomains. NCO then reacts with chain extenders, moisture, or active hydrogen, which promotes chain growth and increases hydrogen bonds, urea bonds, and urethane bonds. PU-rich phases next develop from particles or aggregates into locally connected or crosslinked networks. Finally, this network couples with components such as asphaltenes to form an integrated structure.

### 3.3. Modification Mechanisms of Asphalt Binder Using the Prepolymer Method

PU modification by the prepolymer method introduces an NCO-containing reactive intermediate into the continuous asphalt binder phase, so PU formation, curing, and interfacial bonding occur concurrently within the colloidal system. The mechanism can be considered at three levels. Chain extension, ureation, urethanisation and crosslinking of the prepolymer form a load-bearing network; the in situ PU phase swells, disperses, entangles and rearranges with light fractions, resins and asphaltenes in the asphalt binder; and a small amount of terminal NCO reacts with polar active sites in the asphalt binder, aggregate hydroxyl groups, and moisture or degradation products from aged SBS, forming local interfacial anchoring [31,32,44,60,68,69,70,71,72,73,76,78,79,80,81].

The main chemical reaction between prepolymer and asphalt binder is a nucleophilic reaction of the terminal NCO with active hydrogen. MDI-PEG and MDI-PPG systems show that NCO can react with hydroxyl, amino, and carboxyl groups and with moisture in asphalt binder to form urethane, urea, or amide. FTIR can track consumption of the NCO peak at approximately 2270 cm^−1^, and component analysis links the reaction mainly to polar components such as resins and asphaltenes [68,69,70,71].

**Figure 9:**
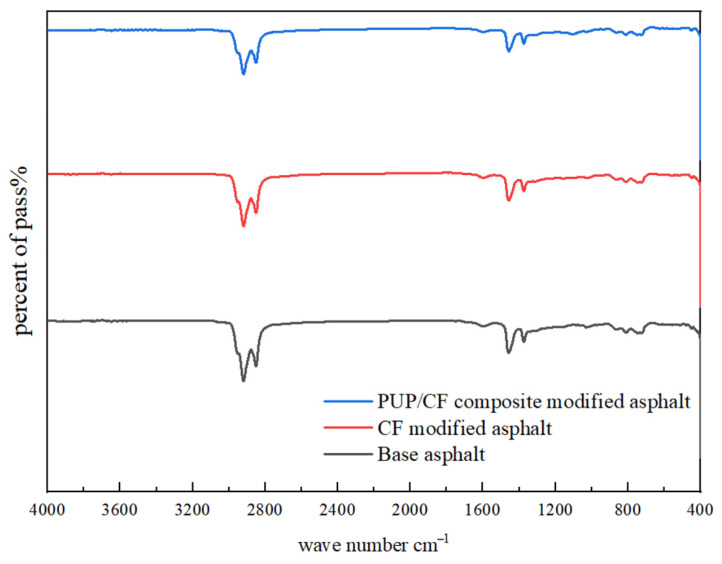
Infrared spectra of base asphalt, CF-modified asphalt, and PUP/CF composite-modified asphalt [78].

In Figure 9, the PUP/CF composite-modified asphalt binder shows stronger absorption near 1595 cm^−1^ than the base asphalt binder. This absorption is associated with the entry of benzene-ring structures from the polyurethane prepolymer into the asphalt binder system through chemical bonding or physical mixing [78]. Moisture participates in post-curing by promoting the conversion of residual NCO to amines and subsequent reactions, which increases the molecular weight of the polymer–asphalt binder system [70]. This behaviour helps explain why many prepolymer-modified asphalt binders continue to evolve after mixing.

Using FTIR, Corbett separation, confocal fluorescence microscopy, and particle-size distribution, Li et al. found that PU generated in situ from PU prepolymer and chain extender in asphalt binder had little effect on the bulk chemistry of the asphalt binder and mainly changed rheological response through physical dispersion and particle suspension [73].

These conclusions are not mutually exclusive. PU can remain predominantly physically dispersed in the bulk binder while limited chemical bonding occurs locally at polar resin/asphaltene domains when residual NCO and accessible active-hydrogen sites are available. Their relative contributions therefore depend on formulation and curing history.

Within the prepolymer, PU and asphalt binder system, the prepolymer first enters the asphalt binder as a flowable, reactive intermediate and then cures into a skeleton enriched in hydrogen bonds. The asphalt binder acts as both the reaction medium and a source of polar sites. Carrera et al. linked prepolymer structure to rheological reinforcement [31]; Yang et al. showed that shear temperature, shear rate, and PUP dosage determine whether this reinforcement becomes a stable structure [67]; and Sun et al. found that PUP, MDBA, and compatibiliser contribute respectively to network formation, chain-extension curing, and compatibilisation stability [60].

Studies of aggregate interfaces further support the role of terminal NCO. Li et al. used PUP to reinforce the asphalt binder–aggregate interface. FTIR, XPS, pull-off, and water-stability tests showed that NCO in PUP can react with hydroxyl groups on the aggregate surface, changing the interface from physical adsorption to chemical-bond-assisted reinforcement and improving adhesion and resistance to moisture damage [81]. This evidence complements the higher surface energy observed in PUPB/FRAP and shows that the prepolymer method modifies the asphalt binder matrix as well as asphalt binder-aggregate, asphalt binder-aged asphalt film, and asphalt binder-reinforcing phase interfaces [76,78,81].

Residual NCO provides a more direct reactive pathway than some conventional adhesion promoters. For example, the coal-tar phenolic-fraction resin PhCR-F raised adhesion to glass from 33% to 87% at 1 wt% and improved retained aggregate coverage after water exposure, but FTIR showed no detectable bulk chemical reaction with bitumen; the effect was attributed mainly to mixing or dissolution and additional weak physicochemical structures [15]. Thus, phenolic promoters primarily strengthen polar interfacial affinity, whereas PUP can additionally form covalent bonds through NCO reactions with hydroxylated mineral surfaces. Both routes improve moisture resistance, but the PU route couples binder-network curing with aggregate-interface anchoring.

The prepolymer method, therefore, uses PU dispersibility, terminal-group reactivity, and post-curing ability to generate PU networks, rearrange asphalt binder phases, and strengthen interfaces in asphalt binder. The process starts with improved mixing and dispersion, proceeds through chain extension or moisture-induced network formation, and then creates local anchoring in polar microdomains of the asphalt binder, aged polymer fragments, aggregates, or fibre interfaces. On this basis, the prepolymer method can be used for base asphalt binder modification, high-RAP-content modification, aged SBS regeneration, interfacial reinforcement, and functional self-healing systems.

## 4. Process-Control Differences in PU-Modified Asphalt Binder and Preparation Advantages of the Prepolymer Method

### 4.1. Differences Among PU-Modified Asphalt Binders Prepared by Different Processes

PU-modified asphalt binders include preformed PU blending, one-step in situ synthesis, the prepolymer method, semi-prepolymer or blocked-prepolymer methods, waterborne PU or emulsified asphalt binder methods, and reactive modification with liquid PU precursors. Yang et al. noted that these routes mainly differ in the order of raw-material addition and in where the PU network is generated within the asphalt binder system [82]. Cong and Zhang further distinguished melt blending, in situ synthesis, and the prepolymer method: melt blending relies on dispersion of prefabricated PU; in situ synthesis directly introduces isocyanate, polyol, and chain extender into the asphalt binder; and the prepolymer method first forms an active-terminal prepolymer that continues to react in the asphalt binder [83].

The preformed PU blending method adds TPU, PU particles, or elastomers to hot asphalt binder as external polymers, in a process similar to conventional SBS or crumb rubber modification. The advantage is that the PU structure has already been defined, and side reactions are limited. The limitation is that performance is governed mainly by melting, swelling, and shear dispersion. If compatibility with the polar components of asphalt binder is insufficient, aggregation or phase separation can occur, and relatively high temperature or strong shear is usually required [83,84]. This method is therefore better suited to physical reinforcement and process simplification.

One-step in situ synthesis directly adds polyol, isocyanate, chain extender, and catalyst to the asphalt binder, allowing PU chain growth and asphalt binder modification to occur at the same time. Yang et al. added PPG, chain extender, and MDI to the asphalt binder at 140–150 degrees C and tuned performance through the isocyanate index and chain extender type. FTIR showed that NCO reacted with both polyol and active functional groups in the asphalt binder, generating urethane and urea bonds in the asphalt binder [82]. The method avoids prepolymer preparation and favours continuous processing, but concurrent multicomponent reactions make the system more complex; moisture, impurities, feeding order, and stirring conditions all affect chain growth, crosslinking, and viscosity growth [82,83].

The prepolymer method first synthesises a PU prepolymer containing NCO or other active terminal groups outside the asphalt binder, then adds it to the asphalt binder as a liquid or low-viscosity reactive modifier. Carrera et al. showed that prepolymer molecular weight and free NCO content affect the viscoelastic response of asphalt binder [31]. Gong et al. compared the CO_2_-based PU prepolymer method with in situ polymerisation and found that both could cure naturally, but their high- and low-temperature responses and glass-transition behaviour differed because chain-growth location and hard-segment proportion differed [44]. The prepolymer method, therefore, shifts part of the PU-chain construction to the prepolymer stage. After the prepolymer enters the asphalt binder, the main processes are dispersion, terminal-group reaction, and network formation.

The semi-prepolymer and blocked-prepolymer methods extend the prepolymer approach. In the semi-prepolymer method, part of the polyol or isocyanate is prereacted before additional components are added to the asphalt binder. In the blocked-prepolymer method, NCO terminal groups are temporarily blocked to reduce activity during storage and before construction. Cong and Zhang blocked a PU prepolymer with methyl ethyl ketone, achieving a blocking ratio of 97.35% and a deblocking temperature of approximately 120 degrees C. After deblocking, NCO continued to react with chain extenders and active components in the asphalt binder to form a crosslinked network [83]. The end-capped PU prepolymer reported by Gong et al. also maintained low activity at room temperature and participated in asphalt binder modification after heating [85]. These methods separate high reactivity from storage and handling requirements by assigning them to different temperature ranges.

**Figure 10:**
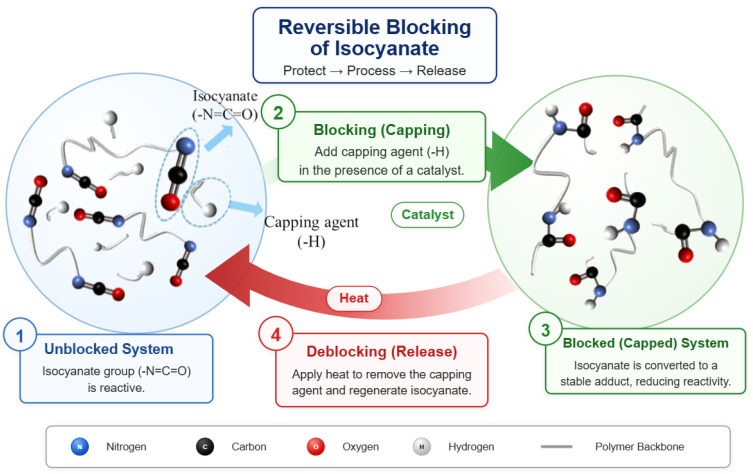
Blocking and deblocking reactions of blocked isocyanates.

Figure 10 shows that the blocked-prepolymer method first masks isocyanate activity with a blocking agent. Thermal deblocking then releases NCO and restores its ability to react with chain extenders or active components in the asphalt binder [83]. This design separates storage from curing and gives reactive PU-modified asphalt binders more controllable processing conditions.

Waterborne PU and waterborne PU asphalt binder routes differ from reactive hot-mix systems. Zhao et al. prepared waterborne PU as particles and added them to the asphalt binder; FTIR showed no obvious new peak, indicating mainly physical blending. At low dosage, storage stability and workability were good, whereas high dosage promoted aggregation and reduced compatibility [84]. Wu et al. also detected no characteristic NCO peak when preparing WPUA and regarded the system as mainly physically mixed. When WPU increased from 30 wt% to 40 wt%, the continuous phase changed from the asphalt binder phase to the PU phase, and mechanical properties changed markedly with phase state [86]. Waterborne routes are therefore more suitable for low-emission, low-temperature film-forming and emulsified asphalt binder applications and should not be conflated with reactive modification using NCO-terminated prepolymers.

As a non-reactive process analogue, Butonal NX4190 cationic styrene–butadiene latex illustrates how a liquid-polymer route can be governed by dosage and residence time rather than terminal-group chemistry. At 448 K, tests with 2–6 wt% latex and 3–9 h blending showed that most changes in penetration, softening point, and elasticity occurred within the first 3 h; 2–4 wt% and 3–4 h were identified as practical optima [87]. The comparison is instructive because both latex and PUP routes require dispersion–viscosity control, but latex modification is dominated by polymer dispersion and film formation, whereas PUP processing couples dispersion with continuing NCO reactions and curing. The Butonal window was established in the laboratory and still requires plant-scale confirmation.

The liquid PU precursor reactive modification method sits between the one-step and prepolymer routes. Li et al. used a liquid PU precursor with isocyanate functional groups to modify the asphalt binder, allowing liquid–liquid mixing at about 145 degrees C. FTIR, SARA, and microscopic analyses showed reactions with polar asphalt binder components and improved compatibility [88]. This approach avoids prolonged melting of solid polymers and lowers the risk of phase separation associated with forming a complete PU network before blending [88]. The main differences among these process routes are summarised in Table 3.

Differences among these processes mainly reflect how reaction extent and dispersion are distributed across stages, which gives each route its own advantages and limitations.

### 4.2. Advantages of the Prepolymer Method

Compared with other methods, the prepolymer method offers three practical advantages. The prepolymer structure can be designed before blending, which reduces uncertainty from concurrent multicomponent reactions. Liquid or low-viscosity prepolymers disperse more easily and limit prolonged high-temperature processing. NCO or other active terminal groups can then continue chain extension, crosslinking, and interfacial connection in the asphalt binder, so physical reinforcement is coupled with chemical modification. The main advantages and underlying mechanisms of the prepolymer method are summarised in Table 4.

Sun et al. provide a field-scale example in which process design was used to bypass, rather than fully solve, storage instability of finished PU-modified binder. For the dry-mix formulation, PU and diluent were set at 6 and 5 wt% of the binder and MOCA at 0.8 wt%; the mixture was produced at 160 °C for 60 s and placed in a 150 m G6911 trial section [89]. Keeping the PU package separate until plant mixing avoids advance production of finished PU-modified binder and reduces the risk of phase segregation and performance degradation during storage and transport. Its feasibility nevertheless depends on accurate on-site metering, rapid dispersion, and sufficient reaction within the short mixing window, while the study reported only as-built quality and identified long-term service monitoring as future work.

## 5. Performance of PU-Modified Asphalt Binder Prepared by the Prepolymer Method and Its Influencing Factors

### 5.1. Analysis of Physical and Rheological Properties and Durability

At the asphalt binder level, across the three conventional indices, the prepolymer method usually decreases penetration and increases the softening point of modified asphalt binder, consistent with higher consistency and better high-temperature stability. In Guo et al.’s PUP/ceramic fibre composite system, the optimised sample showed a penetration decrease from 66.1 to 38.2 and a softening-point increase from 50.8 to 56.2 degrees C, while ductility fell from 30.2 to 20.4 cm [78]. Hao et al.’s HM-PU system also showed a clear increase in softening point, with only a small change in ductility [90].

**Figure 11:**
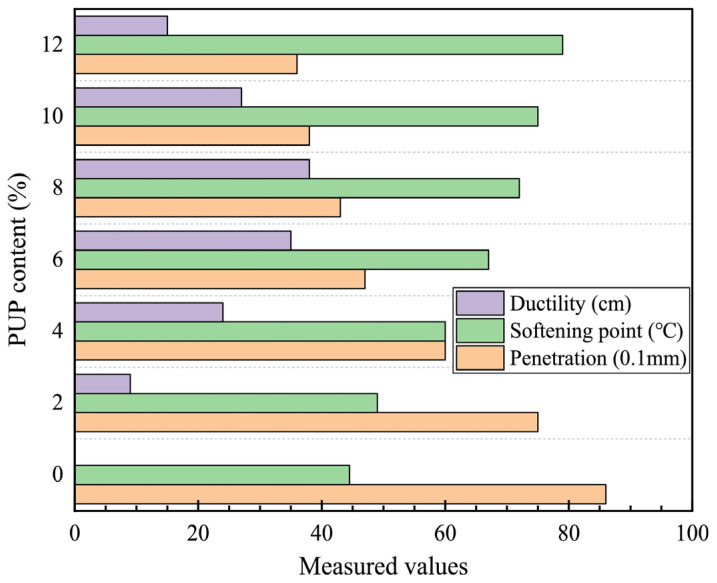
Three basic-index results for PUP-modified asphalt binder at different PUP contents [67].

Figure 11 shows that penetration generally decreased and softening point steadily increased as PUP dosage rose from 0% to 12%. Ductility did not increase monotonically: it was relatively better at intermediate dosages and declined when dosage was excessive [67]. An appropriate PUP dosage can therefore balance high-temperature stability and low-temperature deformability, while excess prepolymer may impair performance.

At the mixture level, mechanical gains are mainly seen in strength, rutting resistance, crack resistance, and resistance to water damage. For warm-mix porous asphalt with 10% prepolymer, after mixing at 150 degrees C and compaction at 130 degrees C, Li et al. reported an ITS stiffness of about 2400 MPa, ravelling loss of about 10%, dynamic stability of 5250 passes/mm, and TSR of about 91%, indicating better performance than conventional porous asphalt [91]. Later water-damage studies found smaller performance losses after freeze–thaw, semicircular bending, ravelling, and Hamburg rutting tests [92]. In Sun et al.’s high-dosage system, tensile strength increased during curing from about 2.45 to 4.09 MPa [60]. Shirzad et al. reported that 5–10% self-healing prepolymer improved healing and crack resistance, whereas 15% reduced strength and cohesion [24].

At the asphalt binder level, the prepolymer method generally increases complex modulus, viscosity, rutting factor, and elastic recovery. Carrera et al. found the greatest improvement when PPG was about 940 and free NCO was 17.4 wt%; after water participation, viscosity in some systems rose by about three orders of magnitude [31]. Li et al.’s PRM modifier could be prepared at about 145 degrees C and improved high-temperature rutting resistance, fatigue resistance, and ageing resistance [88]. Frequency sweeps by Yang et al. showed that 8% PUP retained an elastic advantage over a broad temperature range [67].

In high-temperature service, the elastic skeleton formed through prepolymer curing or chain extension can lower penetration and raise softening point, complex modulus, and rutting factor. In Gong et al.’s CO_2_-based P-PUMA system, resistance to permanent deformation at high temperature continued to improve during natural curing, so the degree of post-curing affected high-temperature performance [44]. At low temperature, Li et al. found that increasing PRM dosage raised BBR stiffness and lowered the m-value; 1.5 wt% improved fracture energy at −12 degrees C, whereas 4 wt% could be detrimental [93]. After 15 days of curing, Gong et al.’s P-PUMA system had a lower stiffness modulus and a higher m-value at −18 degrees C, consistent with better low-temperature creep behaviour [44].

For durability, prepolymer-modified asphalt binder usually shows lower mass loss, a higher retained penetration ratio, and more stable performance after ageing. After RTFOT ageing, Guo et al.’s PUP/ceramic fibre system had lower mass loss than base asphalt binder, a higher retained penetration ratio, and a smaller softening-point increment, which suggests lower sensitivity to short-term thermo-oxidative ageing [78]. Li et al. reported that PRM-modified asphalt binder retained good rheological and fracture properties after ageing [88]. End-capped prepolymer durability is also governed by deblocking temperature: M-PUP can be effectively deblocked at 135 degrees C for 50 min, whereas C-PUP requires 165 degrees C for 35 min, and the higher temperature may intensify thermal ageing of asphalt binder [85]. Reaction temperature and time, therefore, also influence durability.

Comparisons among modification routes show different strengths for the prepolymer method and in situ polymerisation. In the same CO_2_-based system, Gong et al. found that both routes improved high- and low-temperature performance. In situ polymerisation gave stronger high-temperature performance because it produced higher hard-segment content and a higher reaction degree, whereas the prepolymer method produced a lower Tg and better low-temperature performance [44]. The prepolymer route is mainly useful where structural controllability, lower construction temperature, and low-temperature flexibility must be balanced.

### 5.2. Performance-Influencing Factors of PU-Modified Asphalt Binder Prepared by the Prepolymer Method

The prepolymer structure is the main factor governing modified asphalt binder performance, including soft-segment molecular weight, terminal NCO content, end-capping, and chain extender. Carrera et al. showed that higher free NCO does not necessarily maximise rheological enhancement because chain length and terminal-group concentration must also be balanced [31]. Hao et al.’s HM-PU system similarly showed that thermal stability, high- and low-temperature performance, and compatibility were jointly optimised when NCO content and dosage were both 5% and the system was sheared at 150 degrees C and 3500 r/min for 40 min [90]. Structural design, therefore, has to balance reactivity, construction viscosity, phase dispersion, and low-temperature flexibility.

Dosage is the most direct variable, although the optimal range depends on the system. In low-dosage PRM systems, 1.5 wt% can improve low-temperature fracture energy, while excessive dosage increases the risk of embrittlement [93]. Yang et al. recommended a PUP preparation dosage of 6–10%, with 8% PUP giving better high-temperature deformation resistance [67]. The optimised PUP/ceramic fibre composite used 7.4% PUP, 2.1% ceramic fibre, and 40 min shearing, which balanced conventional indices with ageing resistance [78].

Preparation temperature, shear intensity, and reaction time together determine PU dispersion and curing in the asphalt binder. If the temperature is too low, diffusion, deblocking, or chain extension may be incomplete; if the temperature is too high or the holding time too long, asphalt binder ageing and excessive crosslinking may occur. Gong et al. reported suitable deblocking conditions of 135 degrees C for 50 min for M-PUP and 165 degrees C for 35 min for C-PUP, with the latter more likely to induce thermal ageing [85]. Cong and Zhang synthesised MEKO-BPUP with a free NCO content of 0.83%, a blocking rate of 97.35%, and a deblocking temperature of 120 degrees C. Its deblocking, chain-extension, and curing processes increased softening point, tensile strength, elastic recovery, adhesion, and deformation resistance, with better adhesion at 45% content [83].

Chain extenders and compatibilisers can redirect the reaction pathway and change the macroscopic response. After adding a chain extender and compatibiliser, Sun et al. identified an optimal mass ratio of 100:60:15.6:3 for base asphalt binder, PUP, chain extender, and compatibiliser. This system improved elastic recovery, high-temperature resistance to permanent deformation, and post-curing tensile strength [60].

With suitable prepolymer structure, dosage, and processing, the prepolymer method can lower construction temperature and improve high-temperature deformation resistance, fatigue resistance, and water stability. Under some conditions, it can also maintain or improve low-temperature crack resistance. However, excessive NCO reactivity, dosage, temperature, or curing time can increase viscosity, reduce low-temperature relaxation, shorten the construction window, and raise the risk of phase separation at the same time. Preparation schemes, therefore, need to balance these indices for the intended service environment.

### 5.3. Storage-Stability Limitations of Prepolymers and Improvement Methods

The high reactivity of conventional NCO-terminated prepolymers also causes storage-stability problems. Unblocked prepolymers may continue to react with moisture in the air, active asphalt components, or chain extenders, which increases viscosity during storage or mixing. At high dosage, this can affect pumping, mixing, and compaction [60,67,83].

Storage stability in prepolymer-method PU-modified asphalt binder has two aspects. The prepolymer must first remain liquid or low-viscosity and processable before it enters the asphalt binder. After incorporation, it must stay uniformly dispersed and avoid stratification during hot storage, transport, and construction waiting. The first aspect is controlled by NCO reactivity, moisture, temperature, catalyst, and degree of blocking, while the second also depends on dosage, chain extender, compatibiliser, shearing, storage temperature, and curing time. The central problem is balancing reactivity with compatible dispersion in the asphalt binder.

Residual or terminal NCO groups in isocyanate-terminated prepolymers enable later chain extension, crosslinking, and interfacial reactions, but they can also react with moisture or active hydrogen during storage. These reactions form urea bonds, increase molecular weight and viscosity, and may even cause gelation. Gong et al. found that unblocked PUP gradually cured during ambient storage and showed a marked viscosity increase after 6 h; MEKO- or caprolactam-capped PUP remained liquid for long periods and released NCO after thermal deblocking [85].

**Figure 12:**
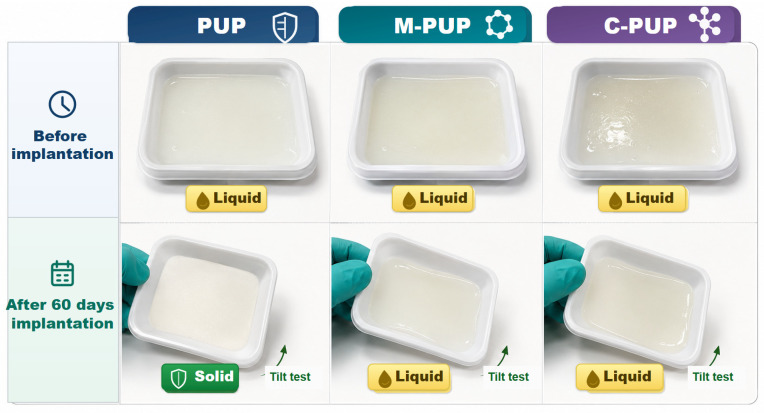
Illustration of appearance and viscosity changes of P-PUP, M-PUP, and C-PUP during storage.

Figure 12 shows that unblocked prepolymers readily undergo rapid viscosity growth and curing, while end-capping markedly delays destabilisation [85]. Storage stability, therefore, depends on temporarily suppressing NCO reactions during storage and reactivating them during preparation.

Prepolymer storage stability is also affected by structural uniformity during synthesis. PEG-IPDI studies showed that temperature, reactant concentration, and catalyst dosage can change the ratio of mono-/difunctional oligomers, which in turn affects later viscosity growth and reaction controllability [63]. Moisture, NCO/OH ratio, temperature, and catalyst should therefore be controlled during synthesis, and MIR/NIR can be used to track NCO content and improve batch stability [64].

Blocked or end-capped prepolymers address these problems. Cong and Zhang used methyl ethyl ketone to block PU prepolymer, which gave NCO low activity at room temperature and allowed it to participate in asphalt binder crosslinking after deblocking at about 120 degrees C [83]. Gong et al. used an end-capped PU prepolymer that remained liquid during room-temperature storage and, after thermal deblocking, could still form a crosslinked system containing urethane, urea-bond, and amide structures [85]. These approaches retain the advantages of pre-built chain segments and continued reaction in asphalt binder while reducing storage limitations.

After the prepolymer enters the asphalt binder, storage stability depends on compatible dispersion and reactive curing acting together. A suitable prepolymer amount can form a dispersed phase or crosslinked network and strengthen interfacial bonding. If the dosage is too high or the reaction is too fast, PU-phase aggregation, a larger softening-point difference, viscosity growth, and a shortened construction window can occur.

Yang et al. prepared PUP-modified asphalt binder with an MDI-PPG prepolymer and found that PUP improved high-temperature performance and low-temperature toughness. When the dosage exceeded about 10%, however, the softening point difference and viscosity growth accelerated markedly, and thermal storage stability decreased [67]. Storage at 163 degrees C also accelerated NCO reactions and agglomeration, while 150 degrees C was closer to actual preparation and construction conditions [67].

**Figure 13:**
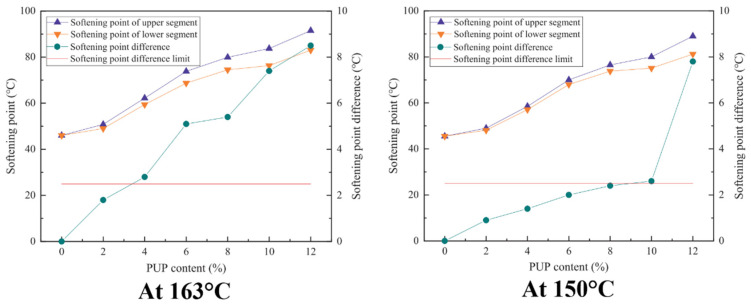
Storage stability and viscosity evolution of PUP-modified asphalt binder at different storage temperatures and PUP contents [67].

Figure 13 shows that PUP dosage and storage temperature jointly control phase separation and viscosity growth. Low dosage maintains flowability and uniformity, while high dosage reduces stability through reaction, phase growth, and enrichment.

In high-dosage PUP binders, chain extenders and compatibilisers are also important. Sun et al. found that increasing PUP and chain-extender contents accelerated viscosity growth and shortened construction retention time. After maleic anhydride was added, the upper–lower layer softening point difference decreased markedly, and 3% compatibiliser was sufficient to meet the segregation criterion [60]. Compatibilisers can therefore suppress PU enrichment and phase separation.

**Figure 14:**
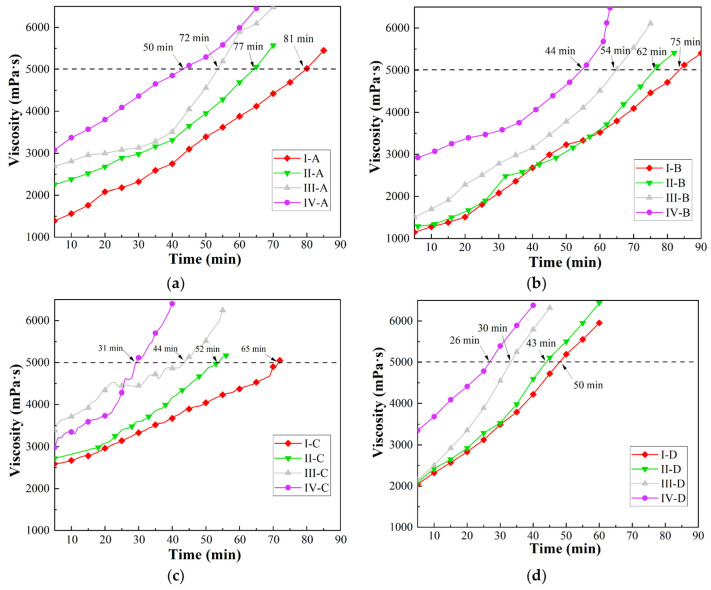
Brookfield viscosity–time curves of PUP-based asphalt binders with different modifier and compatibiliser contents: (**a**) group A; (**b**) group B; (**c**) group C; (**d**) group D [60].

Figure 14 shows that storage stability in PUP systems is closely tied to construction time. Even when the phase-separation index is acceptable, a rapid rise in viscosity over a short period can still restrict pumping, mixing, and compaction.

Blocked prepolymers can extend the workable time of modified asphalt binder. MEKO-BPUP has low activity at room temperature, releases NCO during heating, and forms crosslinked networks with MOCA, maleic anhydride, and active asphalt components. A dosage of 30–45% can produce a relatively uniform two-phase interpenetrating structure, whereas an excessive dosage leads to PU enrichment [83]. The main benefit is that curing is delayed until the mixing and forming stages.

Studies of high-modulus PU prepolymers also show that NCO content and dosage have optimal ranges. Hao et al. found that, when both NCO content and HM-PU dosage were 5%, the PU phase was relatively uniform; further increases reduced dispersibility and storage stability because of excessive crosslinking and particle agglomeration [90]. In the CO_2_-based P-PUMA system reported by Gong et al., the softening point difference after thermal storage was low and decreased as curing time increased, suggesting that ongoing reaction strengthens interphase bonding [44]. Continuous curing, however, also increases modulus and viscosity, so evaluation should consider the softening point difference, viscosity–time curve, curing degree, and microscopic phase morphology together.

Table 5 summarises the main destabilising factors and regulation methods for storage stability in prepolymers and their modified asphalt binder systems.

The storage stability of the prepolymer itself mainly depends on the temporary suppression of NCO reactivity and quality control during synthesis. In prepolymer-method-modified asphalt binder, storage stability depends on the match between reaction rate, compatible dispersion, and the construction window. Blocked prepolymers, low-moisture control, suitable NCO content, reasonable prepolymer dosage, chain-extender ratio, and compatibiliser design are currently the main approaches for improving storage stability in these systems.

## 6. Composite Modification Processes Using Prepolymer PU and Applications in Non-Traditional Asphalt Binder Systems

In practical road materials, prepolymer PU is usually not used only as a single modifier. It can also be introduced into existing polymer-modified asphalt binder, recycled asphalt binder, open-graded or porous asphalt, and interface-reinforcement systems. In these systems, the function of the prepolymer extends beyond improving base asphalt binder performance to reactive coupling, repair of aged structures, stronger interfacial adhesion, and regulation of composite networks.

### 6.1. Application in SBS-Modified Asphalt Binder

SBS-modified asphalt binder is widely used, but its network is dominated by physical dispersion, swelling, and vulcanised structures, and ageing and loading weaken its continuity. PU precursors or prepolymers can react through NCO with polar asphalt components and active groups in degradation products of aged SBS, forming new chemical connections outside the SBS network. Liu et al. found that PRM increased high-temperature PG, MSCR elastic recovery, resistance to permanent deformation, and LAS fatigue life, and reduced contrast between the SBS phase and asphalt phase, which improved colloidal structure and network compatibility [94]. These results suggest that PU precursors can reinforce the continuity and stability of the original SBS-modified asphalt binder network through chemical reactivity.

Regeneration of aged SBS-modified asphalt binder more clearly shows the advantages of prepolymer PU. Conventional rejuvenators mainly replenish light components and have limited ability to repair chain-scission damage in SBS networks. Zheng et al. used PUP synthesised from PTMEG/MDI to regenerate aged SBS mixtures and proposed that it both replenishes low-molecular-weight components and repairs degraded networks. With 4% PUP, the regenerated mixture recovered low-temperature bending strength, maximum bending strain, water-immersed Marshall residual stability, and TSR to levels close to or above those of fresh mixtures [95]. GPC, FTIR, and fluorescence morphology further showed that terminal NCO groups in PUP reacted with active sites such as hydroxyl and carboxyl groups in aged SBS and reconstructed the polymer network [95].

**Figure 15:**
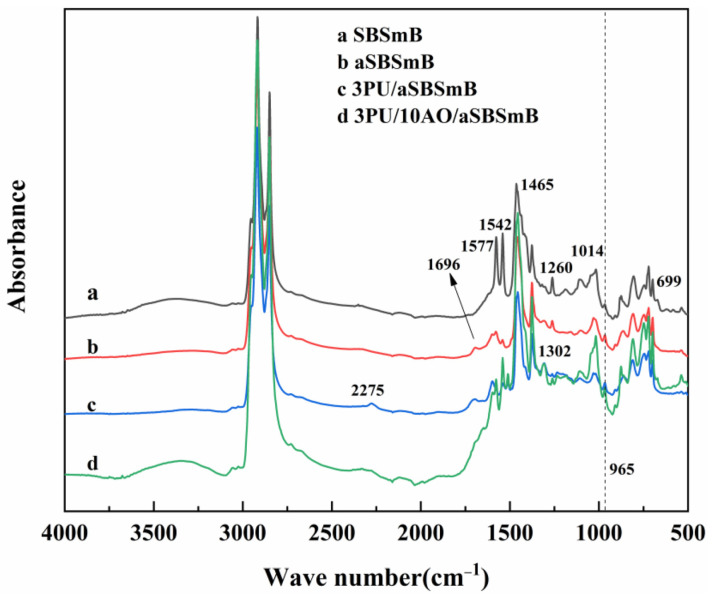
Effect of different rejuvenators on the molecular structure of aSBSmB [79].

The FTIR spectra in Figure 15 show a clear chemically reactive rejuvenating effect of PU on aged SBS-modified asphalt binder [79]. Shu et al. regenerated aged SBS using 3 wt% PUP together with 10 wt% aromatic oil; these components provided structural reconstruction and aromatic-component replenishment, respectively [79]. Hu et al. further treated aged SBS with PU precursor and BUDGE, finding that the carrier-supported method was more favourable than direct addition for improving permanent deformation resistance, water stability, and low-temperature crack resistance [80]. PUP/PU precursor should therefore be used together with light components, flexible reactive agents, or carrier systems to balance SBS network repair with recovery of aged asphalt binder components.

In RAP/FRAP, prepolymer PU also contributes to aged-polymer repair and interfacial adhesion. The PUPB used by Wang et al. for 100% RAP could form urea bonds, amide, and crosslinked structures, improving high-temperature deformation resistance, low-temperature crack resistance, and PUPB-RAP interfacial adhesion/cohesion [76]. In 20% and 40% RAP mixtures, Li et al. found that PUP could react with functional groups in new and aged asphalt binder, increasing binder stiffness, elasticity, and aggregate/RAP interfacial adhesion, which improved high-temperature, water-stability, and low-temperature performance [96]. Together with results for PPB in FRAP, these findings show that prepolymer PU mainly provides reactive connections among aged asphalt binder, aged polymer, and new components [60].

### 6.2. Application in Composite Modification

Besides SBS, prepolymer PU can be compounded with RET, crumb rubber, block copolymers, fibres, and warm-mix additives. RET has good high-temperature performance and storage stability but insufficient low-temperature performance. Xu et al. found that PUP can react chemically with RET-modified asphalt binder, improving RETMA and mixture performance, especially low-temperature performance, and recommended a combination of 1.5% RET and 8–10% PUP [97]. These results suggest that PUP can compensate for the limited low-temperature toughness of RET while maintaining the stability of chemically modified asphalt binder.

SBS/PUP composite systems can be used for high-viscosity, high-elasticity asphalt binder. Tian et al. recommended SBS, PUP, chain extender, and sulphur crosslinker contents of 4%, 5%, 0.5% and 1 per mille, respectively. FTIR confirmed that PUP participated in chemical reactions, and fluorescence images showed smaller SBS particles with a more uniform distribution [98]. MSCR and DSC showed that SBS/PUP-HVEA had lower stress sensitivity and better thermal stability than SBSMA, and met multi-grade high-temperature PG requirements [98]. Together with the SBS-PRM system, these findings indicate that prepolymer/precursor PU can act as a reactive component in SBS composite modification, improving high-temperature deformation resistance, fatigue performance, and phase stability [94,98].

The main limitations of crumb-rubber-modified asphalt binder are insufficient compatibility and storage stability. Liu et al. prepared a physical–chemical composite-modified asphalt binder using CR/PRM and found that the high-temperature performance and fatigue life of CPMA were better than those of systems modified with CR or PRM alone. PRM formed a three-dimensional crosslinked network and improved thermal storage stability, while the flexibility and rebound resilience of CR alleviated low-temperature hardening [99].

Block-copolymer-modified PUP shifts PUP from an additive to a designable composite prepolymer. Jin et al. introduced a hydrogenated styrenic block copolymer into polyether-type PPU to prepare M-PPU, which formed an interpenetrating network in the asphalt binder. At 4%, M-PPU outperformed 5% PPU in softening point, ductility, stripping resistance, surface free energy, and AFM nanomechanics. SEM/AFM showed a more uniform and compact surface, reduced honeycomb structure and roughness, and increased adhesion [100].

**Figure 16:**
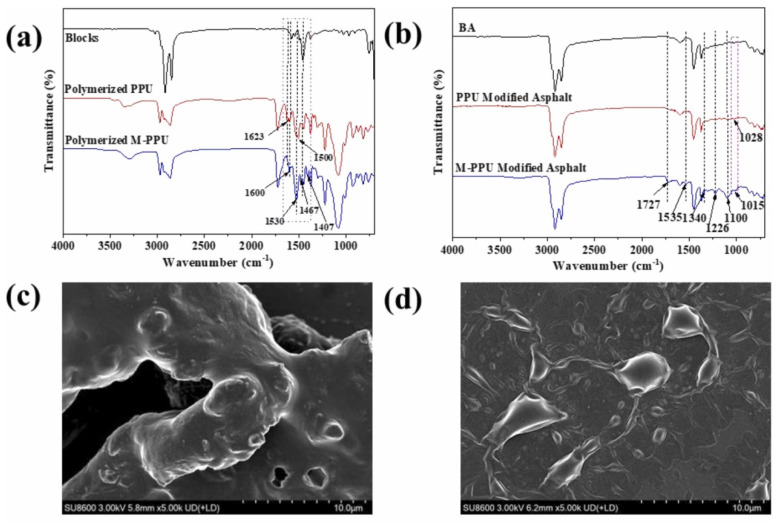
FTIR spectral comparison of modifiers (**a**), FTIR spectral comparison of asphalt samples (**b**), SEM images of PPU modified asphalt (**c**), SEM images of M-PPU modified asphalt (**d**) [100].

**Figure 17:**
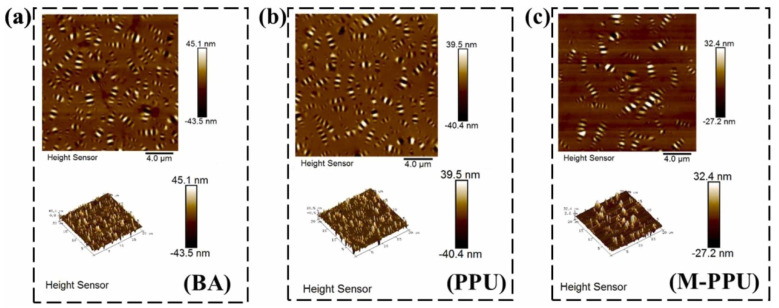
Surface microstructure of BA (**a**), Surface microstructure of PPU modified asphalt (**b**), Surface microstructure of M-PPU modified asphalt (**c**) [100].

Figure 16 and Figure 17 show that M-PPU increases polar structural density through reaction participation of the block copolymer and forms a more continuous, smoother network. Composite modification can therefore complete structural pre-design before the prepolymer enters the asphalt binder, giving it flexible chain segments, a rigid skeleton, and interfacial activity.

Composite modification with functional additives is also an important application direction. Zhao et al. used isocyanate-terminated PUP, BDO, MAH, and Sasobit to prepare warm-mix composite-modified asphalt binder, and identified an optimised process of 135 degrees C, 30 min, 3000 r/min, and 2 h curing; PU improved high-temperature performance, whereas Sasobit improved the viscosity–temperature characteristics in the construction temperature range [101]. After response surface optimisation, the PUP/ceramic fibre composite system reported by Guo et al. showed favourable ageing and rheological, deformation-recovery, and microstructural stability [78]. Prepolymer PU can therefore be combined with warm-mix additives, fibres or particulate reinforcements in designs that target construction viscosity, cracking resistance and toughness, ageing resistance and structural support. Representative applications of prepolymer PU in composite-modified asphalt binders are summarised in Table 6.

### 6.3. Other Applications

Porous asphalt provides drainage and noise-reduction functions, but its open voids make it susceptible to water damage, ravelling, and fatigue. Li et al. prepared warm-mix porous asphalt with 10% PUP, for which the compaction temperature could be reduced to 130 degrees C while requiring compaction energy comparable to that of PG76 porous asphalt compacted at 160 degrees C [91]. Strength development involved two stages: during mixing and compaction, NCO reacted with hydroxyl groups on aggregates to enhance adhesion; after compaction, moisture induced residual PUP to generate urea groups, extend chain segments, and increase cohesion [91]. PUPA showed a Cantabro loss of approximately 10%, a rutting dynamic stability of 5250 passes/mm, and a TSR of 91% after freeze–thaw conditioning, while largely retaining permeability and noise-reduction functions [91].

For water damage, Li et al. further found, using freeze–thaw splitting, SCB, Cantabro loss, Hamburg wheel tracking, and CT scanning, that PUP can mitigate performance degradation after freeze–thaw conditioning, preserve the integrity of the internal void structure, and reduce cracking, ravelling, and rutting deterioration after water exposure [92]. This extends the role of prepolymer PU from the binder to the void skeleton and wet-state structural stability of open-graded mixtures.

Interfacial reinforcement is another application route. Li et al. used PUP to reinforce the asphalt–aggregate interface, demonstrating that residual NCO can react with hydroxyl groups on aggregate surfaces and polar asphalt binder components, strengthening the bond between the asphalt film and mineral aggregates [81]. The M-PPU study by Jin et al. also showed that predictions of water stability based on surface free energy have limitations, whereas AFM adhesion force and modulus distributions better reflect mechanical continuity in the microscopic contact region [100]. Evaluation of these systems should therefore consider asphalt-film coverage, interfacial adhesion, void structure, and retained performance after water exposure together [102].

Recycled asphalt binder, porous asphalt, high-viscosity high-elasticity asphalt binder, and prepolymer–aggregate reactive systems each have clear application orientations. They share the advantage of retaining low-viscosity or liquid-state construction characteristics while reactions continue during mixing, compaction, curing, or early service. For RAP/FRAP, PUP mainly repairs aged asphalt binder and aged polymer structures [76,95,96]; for porous asphalt, PUP mainly enhances adhesion, cohesion, and wet-state structural retention [91,92]; for composite polymer-modified asphalt binder, PUP/PRM mainly improves compatibility, network continuity, and mechanical balance [94,97,98,99,100,101].

Applications of prepolymer PU in other polymer-modified asphalt binders and non-traditional systems can be grouped into three categories. The first is reactive recycling for aged SBS, RAP, and FRAP, in which reactions with aged SBS, resins, asphaltenes, and active groups restore the polymer network, reinforce interfaces, and improve recycling performance [76,79,80,95,96]. The second is composite modification involving SBS, RET, crumb rubber, block copolymers, Sasobit and fibres, where synergy between reactivity and elasticity, warm-mix effects or structural support improves compatibility, deformation resistance, fatigue resistance and ageing resistance [78,94,97,98,99,100,101]. The third is functional application in porous asphalt and interfacial reinforcement, where reactions with aggregate hydroxyl groups, moisture, and polar asphalt binder groups improve adhesion, cohesion, and wet-state durability while maintaining drainage and noise-reduction functions as far as possible [81,91,92]. These studies show that prepolymer-route PU has expanded from improving base asphalt binder performance to repairing aged polymers, enabling high-value use of recycled materials, and designing special-function pavements.

## 7. Conclusions and Recommendations

This review summarises research progress on PU and derivative-modified asphalt binders prepared by the prepolymer method, covering material composition, reaction mechanisms, microstructural evolution, performance changes, storage stability, and multi-scenario applications.

(1) The soft/hard segment structure, urethane/urea bonds, and hydrogen bonding of polyurethane provide flexibility, strength, elastic recovery, and microphase-structure regulation. In asphalt binder systems, however, these capabilities are restricted by several factors, including the SARA fraction ratio, polar functional group distribution, asphaltene aggregation state, and ageing evolution, which ultimately affect prepolymer dispersion, reaction, and interfacial action. Existing studies suggest that prepolymer-route PU modification is not a simple physical blending but a continuous process in which a low-viscosity prepolymer disperses, reacts, cures, and anchors within the asphalt binder, with macroscopic performance governed by both PU network formation and local interfacial connections.
(2) The prepolymer method has clear processing advantages over other preparation methods in PU-modified asphalt binder systems. Compared with blending with preformed PU, it reduces the high-temperature swelling and dispersion difficulties associated with solid polymers; compared with one-step in situ polymerisation, it moves part of the chain-segment construction process to an earlier stage, thereby reducing uncertainty from simultaneous multicomponent reactions. The prepolymer method is therefore a reactive modification strategy that combines prior structural design, low-viscosity dispersion, terminal-group reactions, and post-curing regulation.
(3) An appropriate amount of prepolymer enhances modified asphalt binder performance, whereas excessive addition reduces it. Suitable prepolymer structure, dosage, NCO/OH ratio, free NCO content, chain extender, and processing conditions can improve high-temperature rutting resistance, elastic recovery, fatigue performance, water stability, and interfacial adhesion. However, when reaction activity, dosage, temperature, or curing time exceeds the suitable range, the system becomes prone to rapid viscosity increase, reduced low-temperature relaxation capacity, a narrowed construction window, and increased phase-separation risk. Preparation design for the prepolymer method should therefore balance processing conditions with performance.
(4) Beyond traditional modified asphalt binder systems, prepolymer-route PU applications have expanded into composite modification, reactive recycling, porous asphalt, and interfacial reinforcement. In SBS-modified and aged-SBS recycling systems, PUP can participate in repairing aged polymer networks and connecting old and new structures. In RAP/FRAP systems, reactive terminal groups help improve adhesion among aged asphalt films, aggregate interfaces, and newly added components. In porous asphalt and composite-modified systems, prepolymers act synergistically with warm-mix additives, crumb rubber, fibres, or inorganic particles; this synergy improves deformation resistance, water-damage resistance, fatigue resistance, and structural durability. Prepolymer-route PU is therefore also suitable as a composite-modification auxiliary that can regulate reaction sites, curing processes, and composite partners according to service scenarios.

Future development of prepolymer-route PU-modified asphalt binders should focus on controllable molecular design, multiscale characterisation, and low-carbon recycling. Molecular design, storage stability, and construction adaptability can be improved through soft-segment selection, isocyanate structure, NCO content, and blocking/end-capping strategies. Multidimensional characterisation methods such as FTIR, fluorescence microscopy, AFM, and SEM should be combined with molecular simulation to evaluate PU microstructure, phase formation, and interfacial integration. Further integration with bio-based, waste-derived, warm-mix, self-healing, and high-reclaimed-content technologies may help move prepolymer-route PU-modified asphalt binders from laboratory validation towards low-carbon, long-life, and engineering-ready pavement material systems.

The novelty of this review is its integrated material–reaction–process–performance framework for the prepolymer route. Evidence remains mainly theoretical and laboratory-based; pilot/field validation is limited, and broad industrial implementation has not been established. Lower-temperature processing and longer service life may reduce environmental burdens and life-cycle costs, but these benefits remain unverified because comparable life-cycle emissions, plant energy use, and raw-material and maintenance costs, particularly against SBS, are rarely reported. Future work should quantify these indicators and verify them in monitored full-scale sections.

## Figures and Tables

**Table 1:** Representative chemical structures and names of isocyanates, polyols, and chain extenders commonly used for polyurethane synthesis.

Raw Material Name	Category	Structural Formula and Chemical Name		
Isocyanate	Aromatic isocyanates	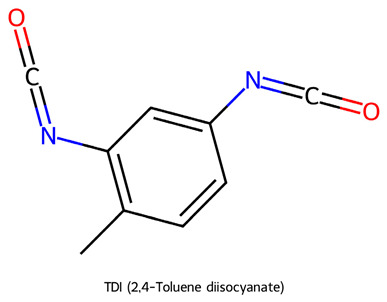 Toluene diisocyanate (TDI)	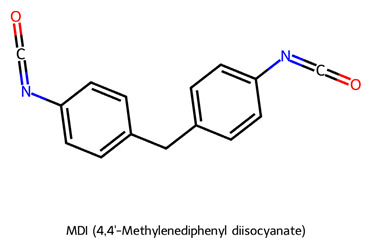 Diphenylmethane diisocyanate (MDI)	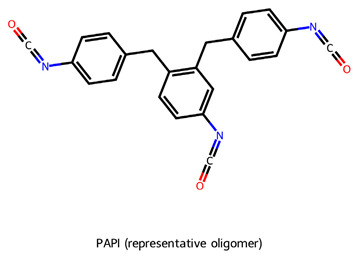 Polymethylene polyphenyl polyisocyanate (PAPI)
	Aliphatic/alicyclic isocyanates	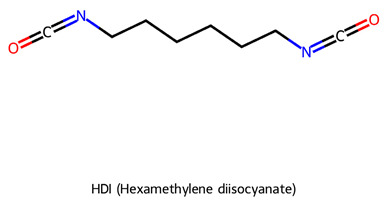 Hexamethylene diisocyanate (HDI)	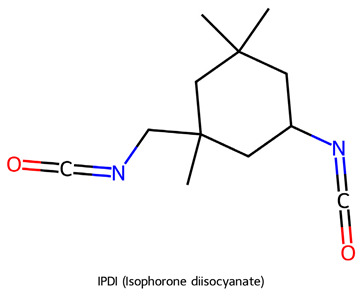 Isophorone diisocyanate (IPDI)	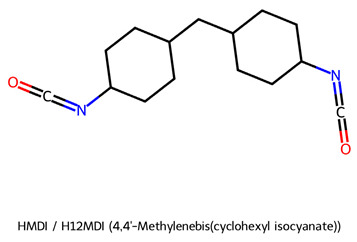 Dicyclohexylmethane diisocyanate (HMDI)
Polyols	Polyether polyols	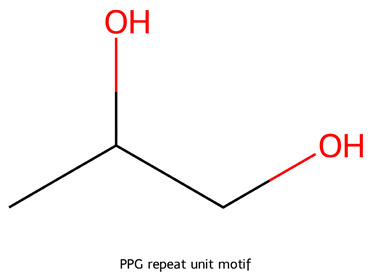 Polypropylene glycol (PPG)	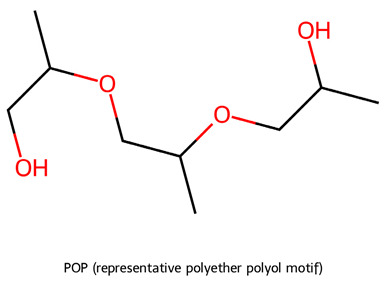 Polyether polyol (POP)	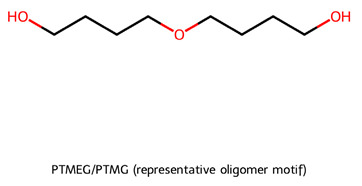 Polytetramethylene ether glycol (PTMEG/PTMG)
	Polyester/polycarbonate polyols	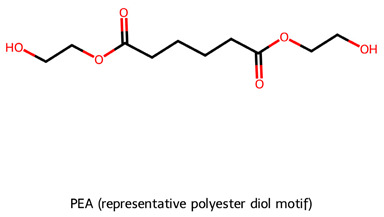 Poly(ethylene adipate) diol (PEA)	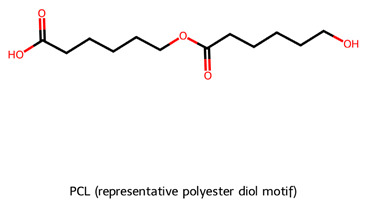 Polycaprolactone diol (PCL)	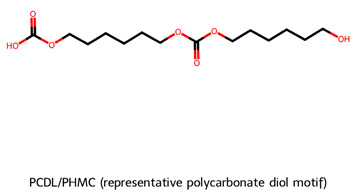 Polycarbonate diol (PCDL/PHMC)
	Other polyols	Hydroxyl-terminated polybutadiene (HTPB)	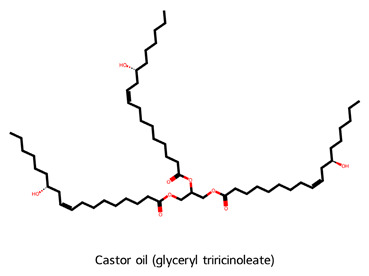 Castor oil polyol (castor oil)	
Chain extenders	Linear small-molecule diols	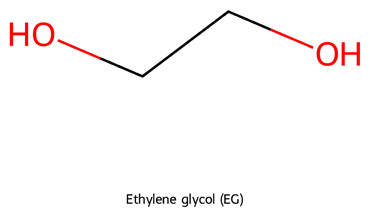 Ethylene glycol (EG)	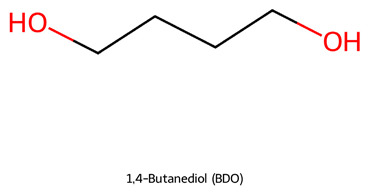 1,4-Butanediol (BDO)	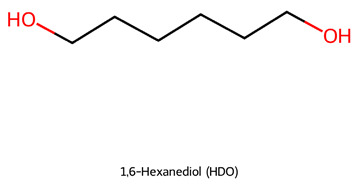 1,6-Hexanediol (HDO)
	Branched/alicyclic chain extenders	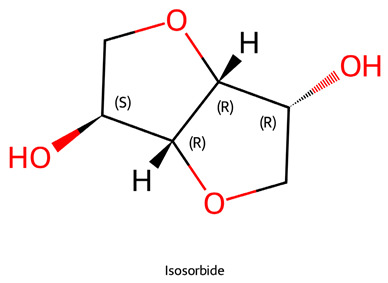 Isosorbide	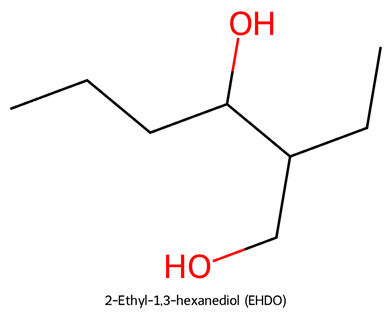 2-Ethyl-1,3-hexanediol (EHDO)	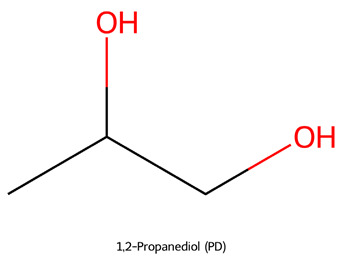 1,2-Propanediol (PD)
	Other/functionalised chain extenders	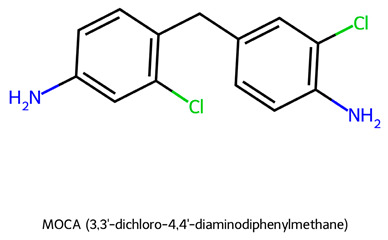 3,3′-Dichloro-4,4′-diaminodiphenylmethane (MOCA)	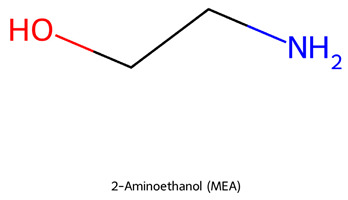 Aminoethanol (MEA)	
		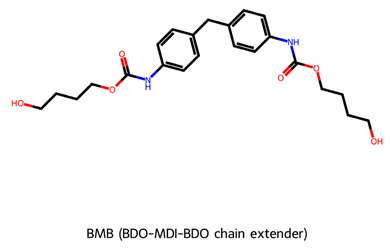 BDO MDI BDO chain extender (BMB)	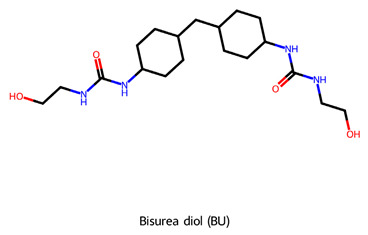 Bisurea diol (BU)	

**Table 2:** Key structural variables in synthesis by the PU prepolymer method and their material significance.

Variable	Effect on Prepolymer/PU Structure	Representative Evidence
NCO/OH ratio and free NCO content	Determine terminal NCO retention, chain-extension/crosslinking capacity, and potential for side reactions; the optimal structure usually depends on the balance between NCO content and molecular size.	In MDI-PPG prepolymers, the combination of 17.4 wt% free NCO and PPG molecular weight of 940 gives a stronger structural-growth effect [31]
Polyol type and molecular weight	Determine soft-segment flexibility, polarity, viscosity, and hard-segment compatibility, and affect NCO accessibility.	PPC-PU and V-PU show that CO_2_-based and vegetable-oil-based soft segments can regulate curing and phase structure [44,66]
Chain extender/crosslinker structure	Determines hard-segment length, hydrogen-bond density, microphase separation, and gelation rate.	BDO, isosorbide, and EHDO alter hard-segment phase separation and crystallisation; MDBA crosslinks with PUP and affects the reaction rate [40,60]
Temperature, catalyst, and moisture	Alter the NCO/OH primary reaction rate and affect NCO–water–amine–urea side reactions and viscosity growth.	MALDI MS and MIR/NIR studies indicate that temperature, catalyst, and moisture are key factors controlling PU prepolymer/polymerisation [63,64]
Shear and residence time	Determine initial dispersion, the contact probability of reactive groups, and the workable viscosity window.	Optimised shear parameters for PUP and the effects of PUP/chain extender on viscosity growth in PPB both show that processing variables are coupled with reaction progress [60,67]

**Table 3:** Main differences among polyurethane-modified asphalt binders prepared by different processes.

Process Route	Addition Mode/Reaction Location	PU-Phase Formation State	Main Advantages	Main Limitations	References
Preformed PU/TPU blending method	Prefabricated PU particles, elastomers, or films are added to the hot asphalt binder and dispersed by shear.	PU is formed before entering the asphalt binder and is mainly physically dispersed in the asphalt binder.	Defined composition, few side reactions, and similarity to conventional polymer modification	Requires high temperature or prolonged shear; insufficient compatibility can cause aggregation/phase separation	[83,84]
One-step in situ synthesis	Polyol, isocyanate, chain extender, and catalyst are directly added to the asphalt binder.	PU chain growth and asphalt binder modification occur simultaneously.	Short process, suitable for continuous operation	Concurrent multicomponent reactions, affected by moisture, impurities, and active groups; narrow processing window	[82,83]
Prepolymer method	Active-terminal PU prepolymer is synthesised first and then introduced into the asphalt binder for curing or chain extension.	Some chain segments form externally, followed by terminal-group reactions and network completion in the asphalt binder.	Controllable reaction; liquid prepolymer disperses readily; adjustable NCO, soft/hard segments, and curing process	Limited storage stability, with the construction window constrained by viscosity growth	[31,44,67]
Semi-prepolymer/blocked-prepolymer method	Partial prereaction or NCO blocking, followed by component addition or thermal deblocking after introduction into the asphalt binder.	Delayed activity, with chain growth/crosslinking retriggered in the asphalt binder.	Improves storage stability and workable time while retaining reactive modification capability	Requires matching of deblocking temperature, time, and ageing-resistance requirements	[83,85]
Waterborne PU/emulsified asphalt binder method	WPU particles, dispersions, or emulsions are mixed with asphalt binder/emulsified asphalt binder.	Dominated by physical compositing and phase-state transition, without reliance on NCO reactions.	Low-emission and low-temperature preparation, suitable for emulsified asphalt binder and cold construction	High dosage can cause phase inversion, aggregation, or reduced storage stability	[84,86]
Liquid PU precursor method	A liquid precursor containing isocyanate functional groups is mixed with hot asphalt binder.	The precursor reacts with polar components and forms PU-related structures.	Liquid–liquid mixing facilitates dispersion, enables lower preparation temperatures, and provides chemical modification	High dosage or excessive reaction increases viscosity and reduces flexibility	[88]
High-content PUP thermosetting asphalt binder	High-dosage PUP, chain extender, and compatibilising components are introduced together into the asphalt binder.	PUP cures to form a continuous or semi-continuous network.	Suitable for high-reclaimed-material or thermosetting binder systems	Viscosity and curing time strongly affect construction timing	[60]

**Table 4:** Main advantages and mechanisms of the prepolymer method for preparing PU-modified asphalt binder.

Advantage Dimension	Core Advantage or Issue	Mechanism and Specific Manifestation	References
Reaction-process controllability	The prepolymer method can separate PU segment construction from asphalt binder modification, improving reaction controllability.	Compared with the one-step method, where the reaction system is complex and relatively uncertain, the prepolymer method first defines the soft/hard segment structure and terminal group content in a comparatively simple system and then introduces the prepolymer into the asphalt binder. The modification stage is therefore mainly focused on dispersion, terminal-group reactions, and the formation of a cured network.	[31,44]
Simplified solid-liquid conversion	The prepolymer method can simplify the softening, melting, or swelling processes required for solid-polymer modification.	Solid PU or TPU usually requires prior softening, melting, or swelling, whereas PU prepolymers are mostly liquid or low-viscosity reactive components that can fully contact hot asphalt binder at lower temperatures. Yang et al. noted that liquid PU feedstocks do not require the high-temperature swelling needed for solid polymers.	[82]
Regulation of physical properties	The prepolymer method preserves chemical modification capability while providing a broader scope for molecular-structure and process regulation.	Prepolymer molecular weight, free NCO content, soft-segment type, hard-segment content, and chain-extension position can all affect the thermal transitions, viscoelasticity, and rheological properties of modified asphalt binder. Dosage, temperature, shear intensity, time, and system scale also jointly control the final properties.	[31,44,67]
Synergistic improvement of interfacial adhesion	PUP can improve adhesion between materials in asphalt mixtures.	PUP can not only disperse within the asphalt binder and form a crosslinked network, but also participate, through reactive terminal groups, in rebinding aged asphalt binder, interfacial interactions, and chemical coupling.	[76,79,80,81]

**Table 5:** Control factors and regulation methods for storage stability of prepolymers and prepolymer-method-modified asphalt binder.

Object	Main Destabilising Factor	Typical Manifestation	Regulation Method	References
Prepolymer	Reaction of NCO with moisture/active hydrogen	Viscosity increase, gelation, and curing	Drying and water exclusion; control NCO/OH	[63,64]
Prepolymer	Excessive activity during storage	Short working time and premature failure	MEKO/caprolactam end-capping; deblock before use	[83,85]
PUP-modified asphalt binder	Excessive PUP dosage	Increased softening point difference and viscosity	Control dosage and avoid exceeding the compatibility threshold	[67]
PUP binder	Excessively fast PUP/chain-extender reaction	Shortened construction retention time	Optimise the ratio and evaluate using viscosity–time behaviour	[60]
PUP binder	Insufficient interfacial interaction	Segregation and PU enrichment	Add an appropriate amount of compatibiliser	[60]
Blocked PUP-modified asphalt binder	Mismatched deblocking temperature	Insufficient curing or thermal ageing	Select low-deblocking-temperature blocking agents and optimise the heating regime	[83,85]
HM-PU-modified asphalt binder	Excessive NCO/PU dosage	Excessive crosslinking and particle agglomeration	Control NCO content and dosage	[90]

**Table 6:** Representative applications of prepolymer polyurethane in composite-modified asphalt binders.

Composite System	Role of Prepolymer/Precursor	Main Performance Changes	Application Relevance
SBS-PRM	PRM reacts with polar asphalt binder components and improves the phase morphology of the SBS network	High-temperature PG, MSCR elastic recovery, and LAS fatigue life are improved, with no obvious deterioration in low-temperature PG [94]	High-temperature, heavy-duty SBS-modified asphalt binder
PUP-regenerated aged SBS	Terminal NCO groups in PUP react with degradation products of aged SBS	Low-temperature bending-tensile properties, TSR, and residual Marshall stability after immersion are close to or exceed freshly mixed levels [95]	High-value recycling of aged SBS
PUP/RET	PUP reacts chemically with RETMA	Improves low-temperature performance and water stability; 1.5% RET with 8–10% PUP is recommended [97]	Compensates for the low-temperature limitation of RET
SBS/PUP-HVEA	PUP participates in reactions and improves SBS dispersion	Storage stability, high-temperature performance, and thermal stability are superior to those of SBSMA [98]	High-viscosity, high-elasticity, and porous asphalt binders
CR/PRM	PRM provides crosslinking, and CR provides flexible rebound	High-temperature performance, fatigue life, and thermal storage stability are superior to those of singly modified systems [99]	Resource utilisation of crumb rubber combined with reactive modification
M-PPU	Block copolymers participate in prepolymer design and form an IPN	The surface becomes denser, and adhesion and stripping resistance are improved [100]	Balances reactivity, flexibility, and interfacial adhesion
PU/Sasobit	PU forms a reactive network, and Sasobit modulates viscosity–temperature behaviour	High-temperature rheology is improved, and viscosity in the construction temperature range is reduced [101]	Warm-mix composite modification
PUP/ceramic fibre	PUP forms a gel network, and fibres provide support	Ageing, rheological, and deformation-recovery performance are improved [78]	Composite enhancement of high-temperature deformation resistance and ageing resistance

## Data Availability

No new data were created or analysed in this study. Data sharing is not applicable to this article.

## References

[1] Perkins S. (2021). Realizing the Roads of the Future. Proc. Natl. Acad. Sci. USA.

[2] Liu Y., Su P., Li M., You Z., Zhao M. (2020). Review on Evolution and Evaluation of Asphalt Pavement Structures and Materials. J. Traffic Transp. Eng. (Engl. Ed.).

[3] Fernández-Gómez W.D., Quintana H.A.R. (2013). A Review of Asphalt and Asphalt Mixture Aging. Ing. Investig..

[4] Lv S., Ju Z., Ge D., Bai Y., Li Z. (2026). Performance Evaluation and Correlation Analysis of UV Aged Asphalt Mixture and Its Extracted Asphalt. Constr. Build. Mater..

[5] Offenbacker D., Mehta Y. (2022). Assessing the Life-Cycle Costs of Pavement Rehabilitation Strategies Used in Long-Term Pavement Performance Program. J. Transp. Eng. B Pavements.

[6] Deng F., Jin J., Chen X., Zhao F. (2023). On the Scheduling of Lifecycle Network-Level Pavement Maintenance: Modelling and Applications. Int. J. Pavement Eng..

[7] Rucker G., Zhang L. (2026). Studying Different Polymer Modified Model Asphalt Using Molecular Dynamics Simulation Methods. Environ. Sci. Pollut. Res. Int..

[8] Li M., Chen X., Cong P., Luo C., Zhu L., Li H., Zhang Y., Chao M., Yan L. (2022). Facile Synthesis of Polyethylene-Modified Asphalt by Chain End-Functionalization. Compos. Commun..

[9] Vargas M.A., Vargas M.A., Sánchez-Sólis A., Manero O. (2013). Asphalt/Polyethylene Blends: Rheological Properties, Microstructure and Viscosity Modeling. Constr. Build. Mater..

[10] Šrámek J., Kozel M., Remek L., Mikolaj J. (2023). Evaluation of Bitumen Modification Using a Fast-Reacting SBS Polymer at a Low Modifier Percentage. Materials.

[11] Porto M., Caputo P., Loise V., Eskandarsefat S., Teltayev B., Oliviero Rossi C. (2019). Bitumen and Bitumen Modification: A Review on Latest Advances. Appl. Sci..

[12] Gunka V., Astakhova O., Hrynchuk Y., Sidun I., Reutskyy V., Mirchuk I., Poliak O. (2024). A Review of Road Bitumen Modification Methods. Part 1—Physical Modification. Chem. Chem. Technol..

[13] Kohut A., Poliak O., Sidun I., Astakhova O., Onyshchenko A., Besaha K., Gunka V. (2025). A Review of Road Bitumen Modification Methods. Part 2—Chemical Modification. Chem. Chem. Technol..

[14] Behnood A. (2019). Application of Rejuvenators to Improve the Rheological and Mechanical Properties of Asphalt Binders and Mixtures: A Review. J. Clean. Prod..

[15] Pyshyev S., Demchuk Y., Poliuzhyn I., Kochubei V. (2022). Obtaining and Use Adhesive Promoters to Bitumen from the Phenolic Fraction of Coal Tar. Int. J. Adhes. Adhes..

[16] Cong L., Yang F., Guo G., Ren M., Shi J., Tan L. (2019). The Use of Polyurethane for Asphalt Pavement Engineering Applications: A State-of-the-Art Review. Constr. Build. Mater..

[17] Wang C., Huang S., Chen Q., Ji X., Duan K. (2023). Materials, Preparation, Performances and Mechanism of Polyurethane Modified Asphalt and Its Mixture: A Systematic Review. J. Road Eng..

[18] Sun G., Kelomae J.M., Li T., Hu M., Cheng L., Liu S. (2026). Valorization of PET Waste via Glycolysis-Derived Polyol into High Performance Polyurethane-Modified Asphalt: Performance Optimization and Sustainability Assessment. Int. J. Adhes. Adhes..

[19] Sun G., Kelomae J.M., Wu H., Cheng L., Cao Z., Wang G. (2026). Sustainable Pavement Binder Application of Glycolyzed Waste Polyethylene Terephthalate to Synthesize High-Performance Polyurethane Modified Bitumen. Constr. Build. Mater..

[20] Zhang Z., Sun J., Jia M., Qi B., Zhang H., Lv W., Mao Z., Chang P., Peng J., Liu Y. (2020). Study on a Thermosetting Polyurethane Modified Asphalt Suitable for Bridge Deck Pavements: Formula and Properties. Constr. Build. Mater..

[21] Jin X., Guo N., You Z., Wang L., Wen Y., Tan Y. (2020). Rheological Properties and Micro-Characteristics of Polyurethane Composite Modified Asphalt. Constr. Build. Mater..

[22] Kong L., Wang Z., Su S., Yue J., Luo W., Zhou S., Ren D., Ai C. (2024). Exploring the Interplay between Thermo-Oxidative Degradation and Asphalt Aging in Thermoplastic Polyurethane-Modified Asphalt: Mechanisms, Properties, and Performance Evolution. Constr. Build. Mater..

[23] Long K., Huang C., Yang Y., Qu C., Huang H., Ai C., Yan C. (2025). Investigation of the Rheological Properties and Aging Performance of Rock Asphalt/Thermoplastic Polyurethane Composite Modified Asphalt. Constr. Build. Mater..

[24] Shirzad S., Hassan M., Mohammad L.N. (2020). Rheological and Mechanical Evaluation of Polyurethane Prepolymer-Modified Asphalt Mixture with Self-Healing Abilities. J. Mater. Civ. Eng..

[25] Huang T., Zhang Z., Wang L., Sun J., Wang Z., Liu H., Chen L. (2022). Study on the Compatibility between Polyurethane and Asphalt Based on Experiment and Molecular Dynamics Simulation. Case Stud. Constr. Mater..

[26] Zhang Z., Wang Z., Zhang S., Liu H., Guo Y., Liu X., Tian P., Yang Y., Xia J. (2024). Experimental Studies and Molecular Dynamics Simulation of the Compatibility between Thermoplastic Polyurethane Elastomer (TPU) and Asphalt. Constr. Build. Mater..

[27] Zhang M., Hu Z., Zhao J., Li H., Zhang J., Lyu L., Wang X., Niu Z., Cai J., Pei J. (2024). Components Optimization of Polyurethane-Modified Asphalt Binder towards Compatibility: Insight from Molecular Dynamics Simulations. Constr. Build. Mater..

[28] Jia M., Zhang Z., Liu H., Peng B., Zhang H., Lv W., Zhang Q., Mao Z. (2019). The Synergistic Effect of Organic Montmorillonite and Thermoplastic Polyurethane on Properties of Asphalt Binder. Constr. Build. Mater..

[29] Ji H., He D., Li B., Lu G., Wang C. (2022). Evaluation of Rheological and Anti-Aging Properties of TPU/Nano-TiO_2_ Composite-Modified Asphalt Binder. Materials.

[30] Zhang Z., Wei Y., Liu X., Guo Y., Liu H., Sun J., Yu X., Kan S. (2023). Combined Modification of Asphalt with Organic Attapulgite (OATT) and Polyurethane (PU): Preparation, Properties and Modification Mechanisms. Constr. Build. Mater..

[31] Carrera V., Cuadri A.A., García-Morales M., Partal P. (2014). Influence of the Prepolymer Molecular Weight and Free Isocyanate Content on the Rheology of Polyurethane Modified Bitumens. Eur. Polym. J..

[32] Cuadri A.A., García-Morales M., Navarro F.J., Partal P. (2014). Processing of Bitumens Modified by a Bio-Oil-Derived Polyurethane. Fuel.

[33] Xia L., Zhang H., Cao D., Guo Y. (2016). Study on Property of Castor Oil Based Polyurethane Modified Asphalt. J. Highw. Transp. Res. Dev..

[34] Zhang Z., Sun J., Jia M., Ban X., Wang L., Chen L., Huang T., Liu H. (2021). Effects of Polyurethane Thermoplastic Elastomer on Properties of Asphalt Binder and Asphalt Mixture. J. Mater. Civ. Eng..

[35] Li Z., Yang F., Yuan J., Cong L., Yu M. (2021). Study on Preparation and Pavement Performance of Polyurethane Modified Asphalt Based on In-Situ Synthesis Method. Constr. Build. Mater..

[36] Cuadri A.A., García-Morales M., Navarro F.J., Partal P. (2014). Effect of Transesterification Degree and Post-Treatment on the in-Service Performance of NCO-Functionalized Vegetable Oil Bituminous Products. Chem. Eng. Sci..

[37] Gholami M., Haddadi-Asl V., Jouibari I.S. (2022). A Review on Microphase Separation Measurement Techniques for Polyurethanes. J. Plast. Film Sheeting.

[38] Santamaria-Echart A., Fernandes I., Barreiro F., Corcuera M.A., Eceiza A. (2021). Advances in Waterborne Polyurethane and Polyurethane-Urea Dispersions and Their Eco-Friendly Derivatives: A Review. Polymers.

[39] Li Y., Jin Y., Fan W., Zhou R. (2022). A Review on Room-Temperature Self-Healing Polyurethane: Synthesis, Self-Healing Mechanism and Application. J. Leather Sci. Eng..

[40] Gallu R., Méchin F., Gérard J.-F., Dalmas F. (2022). Influence of the Chain Extender of a Segmented Polyurethane on the Properties of Polyurethane-Modified Asphalt Blends. Constr. Build. Mater..

[41] Cong P., Liu C., Han Z., Zhao Y. (2023). A Comprehensive Review on Polyurethane Modified Asphalt: Mechanism, Characterization and Prospect. J. Road Eng..

[42] Wang H., Cao L., Wang X., Lang X., Cong W., Han L., Zhang H., Zhou H., Sun J., Zong C. (2024). Effects of Isocyanate Structure on the Properties of Polyurethane: Synthesis, Performance, and Self-Healing Characteristics. Polymers.

[43] Liu H., Zhang Z., Zhang S., Chang P., Liang Y., Wang Z., Ban X., Guo Y., Liu X. (2024). Study on the Effect of Soft Segment Length on the Performance of Polyether-Based Polyurethane Modified Asphalt. Int. J. Adhes. Adhes..

[44] Gong X., Liu Q., Wan P., Chen S., Wang H., Wu J., Wu S. (2023). A Comparative Study of the Properties CO_2_-Based Polyurethane Modified Asphalts Prepared by Prepolymer and in-Situ Polymerization Methods. Constr. Build. Mater..

[45] Huang G., Yang T., He Z., Yu L., Xiao H. (2022). Polyurethane as a Modifier for Road Asphalt: A Literature Review. Constr. Build. Mater..

[46] Yang C., Xie J., Wu S., Amirkhanian S., Zhou X., Ye Q., Yang D., Hu R. (2020). Investigation of Physicochemical and Rheological Properties of SARA Components Separated from Bitumen. Constr. Build. Mater..

[47] Werkovits S., Bacher M., Theiner J., Rosenau T., Grothe H. (2022). Multi-Spectroscopic Characterization of Bitumen and Its Polarity-Based Fractions. Constr. Build. Mater..

[48] Li F., Wang Y., Miljković M., Chan K.M. (2022). Changes in the Nanoscale Asphaltene Particles and Relaxation Spectra of Asphalt Binders during Aging and Rejuvenation. Mater. Des..

[49] Xiao X., Wang J., Wang T., Amirkhanian S.N., Xiao F. (2024). Linear Visco-Elasticity of Asphalt in View of Proportion and Polarity of SARA Fractions. Fuel.

[50] Mirwald J., Werkovits S., Camargo I., Maschauer D., Hofko B., Grothe H. (2020). Investigating Bitumen Long-Term-Ageing in the Laboratory by Spectroscopic Analysis of the SARA Fractions. Constr. Build. Mater..

[51] Hu Z., Zhang H., Wang S., Xu T. (2020). Thermal-Oxidative Aging Mechanism of Asphalt Binder Based on Isothermal Thermal Analysis at the SARA Level. Constr. Build. Mater..

[52] Camargo I.G.d.N., Hofko B., Mirwald J., Grothe H. (2020). Effect of Thermal and Oxidative Aging on Asphalt Binders Rheology and Chemical Composition. Materials.

[53] Pipintakos G., Vincent Ching H.Y., Soenen H., Sjövall P., Mühlich U., Van Doorslaer S., Varveri A., Van den bergh W., Lu X. (2020). Experimental Investigation of the Oxidative Ageing Mechanisms in Bitumen. Constr. Build. Mater..

[54] Pipintakos G., Soenen H., Ching H.Y.V., Velde C.V., Doorslaer S.V., Lemière F., Varveri A., Van den bergh W. (2021). Exploring the Oxidative Mechanisms of Bitumen after Laboratory Short- and Long-Term Ageing. Constr. Build. Mater..

[55] Lu X., Soenen H., Sjövall P., Pipintakos G. (2021). Analysis of Asphaltenes and Maltenes before and after Long-Term Aging of Bitumen. Fuel.

[56] Bruneau L., Tisse S., Michon L., Cardinael P. (2023). Evaluation of Asphalt Aging Using Multivariate Analysis Applied to Saturates, Aromatics, Resins, and Asphaltene Determinator Data. ACS Omega.

[57] Werkovits S., Bacher M., Mirwald J., Theiner J., Rosenau T., Hofko B., Grothe H. (2023). The Impact of Field Ageing on Molecular Structure and Chemistry of Bitumen. Fuel.

[58] Li X., Dong Z., Zhang J., Xu S., Wu W., Liu J. (2023). Research on the Application of Polyurethane Modified Asphalt and Mixture. Mater. Sci..

[59] Yang F., Cong L., Li Z., Yuan J., Guo G., Tan L. (2022). Study on Preparation and Performance of a Thermosetting Polyurethane Modified Asphalt Binder for Bridge Deck Pavements. Constr. Build. Mater..

[60] Sun M., Wang J., Sun H., Hong B. (2023). Feasibility Analysis of Polyurethane-Prepolymer-Modified Bitumen Used for Fully Reclaimed Asphalt Pavement (FRAP). Materials.

[61] Yang F., Gong H., Cong L., Shi J., Guo G., Mei Z. (2022). Investigating on Polymerization Process and Interaction Mechanism of Thermosetting Polyurethane Modified Asphalt. Constr. Build. Mater..

[62] Jin X., Sun S., Guo N., Huang S., You Z., Tan Y. (2021). Influence on Polyurethane Synthesis Parameters upon the Performance of Base Asphalt. Front. Mater..

[63] Blaj D.-A., Diaconu A.-D., Harabagiu V., Peptu C. (2023). Polyethylene Glycol-Isophorone Diisocyanate Polyurethane Prepolymers Tailored Using MALDI MS. Materials.

[64] Fage A.M., Backhaus C.A., Becker W., Lorenz G., Lorenz A., Rebner K., Henning F. (2025). Mid- and near-Infrared Spectroscopies for Quantitative Tracking of Isocyanate Content: Streamlined Development of Monitoring Tools for Reactive Extrusion Synthesis of Specialty Polyurethanes. Polym. Test..

[65] Zhou T., Liu R., Xie S., Fini E., Dong Z., You L. (2026). Disaggregation Mechanisms of Asphaltenes Induced by Castor-Based Bio-Oil: A Molecular Dynamics and Rheological Study. Fuel.

[66] Xia L., Cao D., Zhang H. (2023). Rheological and Aging Properties of Vegetable Oil-Based Polyurethane (V-PU) Modified Asphalt. Polymers.

[67] Yang J., Yin X., Fang Y. (2025). Preparation Process Optimization of Polyurethane Prepolymer Modified Asphalt Binder Based on Response Surface Methodology and Its Rheological Behaviors. J. Appl. Polym. Sci..

[68] Navarro F.J., Partal P., García-Morales M., Martinez-Boza F.J., Gallegos C. (2007). Bitumen Modification with a Low-Molecular-Weight Reactive Isocyanate-Terminated Polymer. Fuel.

[69] Martín-Alfonso M.J., Partal P., Navarro F.J., García-Morales M., Gallegos C. (2008). Use of a MDI-Functionalized Reactive Polymer for the Manufacture of Modified Bitumen with Enhanced Properties for Roofing Applications. Eur. Polym. J..

[70] Martín-Alfonso M.J., Partal P., Navarro F.J., García-Morales M., Gallegos C. (2008). Role of Water in the Development of New Isocyanate-Based Bituminous Products. Ind. Eng. Chem. Res..

[71] Martín-Alfonso M.J., Partal P., Navarro F.J., García-Morales M., Bordado J.C.M., Diogo A.C. (2009). Effect of Processing Temperature on the Bitumen/MDI-PEG Reactivity. Fuel Process. Technol..

[72] Carrera V., Partal P., García-Morales M., Gallegos C., Páez A. (2009). Influence of Bitumen Colloidal Nature on the Design of Isocyanate-Based Bituminous Products with Enhanced Rheological Properties. Ind. Eng. Chem. Res..

[73] Li R., Zhu X., Wang Y. (2024). Investigation of the Physicochemical Interactions and Modification Effects of Polyurethane on Asphalt Binder. Transp. Res. Rec. J. Transp. Res. Board.

[74] Ban X., Zhang Z., Chang P., Zhang S., Liu H., Liang Y., Chen Y. (2023). The Performance and Distribution of Polyurethane-Modified Asphalt That Exhibits Different Molecular Weights. Sustainability.

[75] Zhuang W., Ding T., Pang C., Jiao X., Geng L., Sun M. (2025). Mechanical Properties and Modification Mechanism of Thermosetting Polyurethane-Modified Asphalt. Coatings.

[76] Wang J., Hong B., Wang D., Liu W., Tan S., Lin J., Li T. (2025). Performance and Modification Mechanism Investigation of Polyurethane Prepolymer System Modified Bitumen for 100% Reclaimed Asphalt Pavement (RAP) Application. Constr. Build. Mater..

[77] Shirzad S., Idris I.I., Hassan M., Mohammad L.N. (2025). Self-Healing Capability and Mechanical Properties of Asphalt Mixtures Prepared with Light-Activated Polyurethane Prepolymer Modified Asphalt Binder. Transp. Res. Rec..

[78] Guo T., Guo X., Chen Y., Fang C., Yang J., Li Z., Feng J., Huang H., Li Z., Chen H. (2025). A Study on the Performance of Gel-Based Polyurethane Prepolymer/Ceramic Fiber Composite-Modified Asphalt. Gels.

[79] Shu S., Chen G., Yan J., Li Z., Shen W., Gong K., Luo Y. (2023). Combined Use of Polyurethane Prepolymer and Aromatic Oil in Physicochemical Rejuvenation of Aged SBS Modified Bitumen for Performance Recovery. Polymers.

[80] Hu J., Jiang X., Shen W., Sreeram A., Ren H., Xu X. (2024). Innovative Use of Polyurethane Precursor to Facilitate the Reaction-Rejuvenation of Aged SBS-Modified Asphalt. J. Mater. Civ. Eng..

[81] Li G., Wang Y., Liu H., Li R., Lu G., Chen R., Leng Z. (2025). Enhancement of the Interface Bonding between Asphalt Binder and Aggregate Using Polyurethane Prepolymer as a Chemical Modifier. Int. J. Pavement Eng..

[82] Yang T., He Z., Huang G., Zhao Y., Fu J., Xiang H., Zhou Y. (2023). Study on Materials Composition and Process Parameters of Polyurethane-Modified Asphalt Synthesized in-Situ by the One-Shot Process. Constr. Build. Mater..

[83] Cong P., Zhang X. (2025). Synthesis and Performance of Methyl Ethyl Ketone Oxime Blocked Polyurethane Prepolymer as Modifier for Asphalt Binder. Constr. Build. Mater..

[84] Zhao Y., Gong X., Liu Q. (2023). Research on Rheological Properties and Modification Mechanism of Waterborne Polyurethane Modified Bitumen. Constr. Build. Mater..

[85] Gong X., Liu Q., Liu X., Wan P., Jiang P., Chen S., Wang H., Wu S. (2023). Green Synthesis of End-Capped Polyurethane Prepolymer with High Storage Stability and Its Effects on Bitumen Properties. Constr. Build. Mater..

[86] Wu C., Yang H., Cui X., Chen Y., Xi Z., Cai J., Zhang J., Xie H. (2024). Performance and Morphology of Waterborne Polyurethane Asphalt in the Vicinity of Phase Inversion. Materials.

[87] Onyshchenko A., Lisnevskyi R., Viesich I., Poliak O., Rybchynskyi S., Shyshkin E. (2023). Study on the Effect of Butonal NX4190 Polymer Latex on the Properties of Bitumen Binder and Asphalt Concrete. Chem. Chem. Technol..

[88] Li T., Carreño Gómez N.H., Lu G., Liang D., Wang D., Oeser M. (2021). Use of Polyurethane Precursor–Based Modifier as an Eco-Friendly Approach to Improve Performance of Asphalt. J. Transp. Eng. B Pavements.

[89] Sun J., Zhang S., Liu Y., Zhang Z., Liu X., Zhang Z., Ban X. (2023). Construction Technology and Pavement Performance of Dry-Mix Polyurethane Modified Asphalt Mixtures: A Case Study. Sustainability.

[90] Hao J., Xu Q., Lv Z., Rui Z., Ding Z., Di E. (2026). Study on the Preparation and Mechanism of High–Modulus Polyurethane Prepolymer (HM–PU)–Modified Bitumen. J. Compos. Sci..

[91] Li G., Kan J., Liu H., Leng Z., Lu G., Li D., Zou F., Yang B. (2024). A Novel High-Performance Warm-Mix Porous Asphalt Prepared with Polyurethane Prepolymer: Strength Development Mechanism and Mechanical and Functional Performance. J. Clean. Prod..

[92] Li G., Niu K., Yang B., Lu G., Han M., Zou F., Leng Z. (2026). Mitigating Moisture-Induced Damage in Porous Asphalt via Polyurethane Prepolymer Modification: Performance Investigation and Void Structure Assessment. Clean. Mater..

[93] Li T., Guo Z., Liang D., Luo S., Zhang Y., Hong B., Lu G., Wang D., Oeser M. (2022). Chemical and Physical Effects of Polyurethane-Precursor-Based Reactive Modifier on the Low-Temperature Performance of Bitumen. Constr. Build. Mater..

[94] Liu J., Song L., Xu J., Li T., Fan Z., Tan Y., Wang D. (2026). Performance Enhancement of SBS Modified Asphalt Using Polyurethane Precursor-Based Reactive Modifiers. Constr. Build. Mater..

[95] Zheng W., Liu Q., Ren S., Wu S., Gong X., Wang H., Lu Z. (2026). Reactive Repair of Degraded SBS Structure in Aged SBS-Modified Asphalt Mixtures via Polyurethane Prepolymer: From Molecular-Level Chemo-Rheological Mechanisms to Macroscopic Performance Recovery. Constr. Build. Mater..

[96] Li R., Ma X., Chen J., Tang N., Zhu H., Leng Z., Tan Z. (2026). Performance Enhancement of Recycled Asphalt Mixtures Using Polyurethane Prepolymer. Constr. Build. Mater..

[97] Xu C., Zhang Z., Zhao F., Liu F., Wang J. (2019). Improving the Performance of RET Modified Asphalt with the Addition of Polyurethane Prepolymer (PUP). Constr. Build. Mater..

[98] Tian Z., Zhang Z., Zhang K., Huang S., Luo Y. (2020). Preparation and Properties of High Viscosity and Elasticity Asphalt by Styrene–Butadiene–Styrene/Polyurethane Prepolymer Composite Modification. J. Appl. Polym. Sci..

[99] Liu J., Fan Z., Wang D., Li T., Yang Q., Wang J., Lu G. (2024). Study on the Performance and Mechanism of Physicochemically Modified Asphalt Based on Spatial Crosslinking by the Crumb Rubber and Polyurethane Precursor. Int. J. Pavement Eng..

[100] Jin X., Li D., Gong M., Jiao B., Sun B., Yang Y., Yang Y., Zhang J. (2025). Study on the Intrinsic Mechanism and Adhesion Performance Mapping of Block Copolymer Modified Polyurethane Prepolymer (M-PPU) Modified Asphalt. Constr. Build. Mater..

[101] Zhao Y., Lin M., Liu B., Zhou H., Zeng S., Mei Y. (2026). Performance Optimization and Synergistic Mechanism of Polyurethane/Sasobit Composite-Modified Asphalt with Different Prepolymer Proportions. Constr. Build. Mater..

[102] Zhou T., Wan S., Dong Z., Xie S., You L., Jin C. (2026). Physicochemical Mechanisms of Asphalt–Aggregate Interface Adhesion: Effects of Mineral Composition and Surface Energy. Constr. Build. Mater..

